# Hierarchical Assembly of a Micro‐ and Macroporous Hydrogen‐Bonded Organic Framework with Tailored Single‐Crystal Size

**DOI:** 10.1002/anie.202208677

**Published:** 2022-10-19

**Authors:** Christopher A. Halliwell, Sandra E. Dann, Jesus Ferrando‐Soria, Felix Plasser, Keith Yendall, Enrique V. Ramos‐Fernandez, Goran T. Vladisavljević, Mark R. J. Elsegood, Antonio Fernandez

**Affiliations:** ^1^ Chemistry Department School of Science Loughborough University Loughborough LE11 3TU UK; ^2^ School of Aeronautical Automotive Chemical and Materials Engineering AACME) Loughborough University Loughborough LE11 3TU UK; ^3^ ICMol University of Valencia Valencia 46980 Spain; ^4^ Laboratorio de Materiales Avanzados Departamento de Química Inorgánica-Instituto Universitario de Materiales de Alicante University of Alicante Alicante E-03080 Spain

**Keywords:** Crystal Growth, Hierarchical Assembly, Hydrogen-Bonded Organic Frameworks, Macropores, Micropores

## Abstract

Porous organic molecular materials represent an emergent field of research in Chemistry and Materials Science due to their unique combination of properties. To enhance their performance and expand the number of applications, the incorporation of hierarchical porosity is required, as exclusive microporosity entails several limitations. However, the integration of macropores in porous organic molecular materials is still an outstanding challenge. Herein, we report the first example of a hydrogen‐bonded organic framework (**MM‐TPY**) with hierarchical skeletal morphology, containing stable micro‐ and macroporosity. The crystal size, from micro to centimetre scale, can be controlled in a single step without using additives or templates. The mechanism of assembly during the crystal formation is compatible with a skeletal crystal growth. As proof of concept, we employed the hierarchical porosity as a platform for the dual, sequential and selective co‐recognition of molecular species and microparticles.

## Graphical abstract

The first example of a crystal of a porous organic material based on a hydrogen‐bonded organic framework with hierarchical micro and macroporosity is reported. During the skeletal growth, the crystal size can be easily tailored from micro to centimetre scale while preserving the hierarchical porosity. We used the hierarchical porosity for the sequential co‐recognition of microparticles and molecular species.

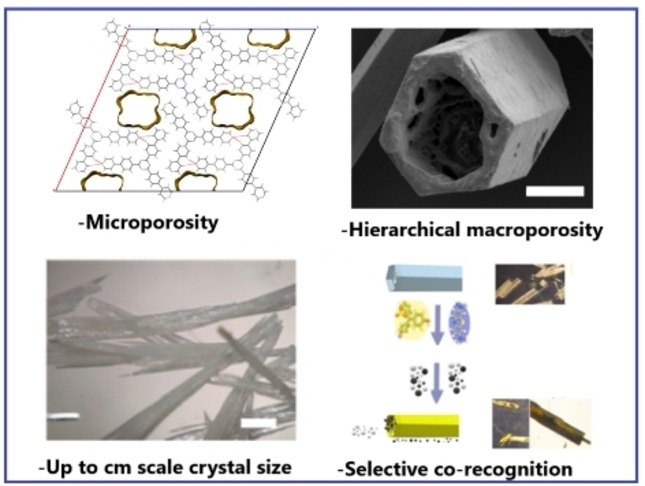

## Introduction

Porous organic molecular materials can be considered as a new type of porous solids characterised by frameworks held together mainly by non‐covalent interactions between their discrete molecular components.^[1]^ Their fundamental molecular nature is translated in a unique combination of properties such as high crystallinity and flexibility, lower density, solution processability^[2]^ and self‐healing nature. These properties, complementary to those exhibited by continuous frameworks, such as metal–organic frameworks (MOFs)^[3]^ and covalent organic frameworks (COFs),^[4]^ make porous molecular materials ideal candidates for advanced applications.[^2^, ^5^] Different families of porous molecular materials have been reported thus far, including porous organic salts,^[6]^ porous organic cages,^[7]^ C−H⋅⋅⋅π microporous crystals,^[8]^ π‐organic frameworks,^[9]^ supramolecular organic frameworks (SOFs),^[10]^ halogen‐bonded organic framework (XOF),^[11]^ intrinsically porous molecular materials (IPMs)^[12]^ and hydrogen‐bonded organic frameworks (HOFs).^[13]^ Despite of the clear progress made, and the great research momentum in the field, outstanding challenges remain, and need to be addressed if the full potential of these materials is to be uncovered. One of these challenges is the introduction of higher levels of hierarchical porosity beyond microporosity, due to the several limitations that microporosity entails, such as the limited access of large species into the pores and an overall slow mass transport, which hampers the expansion and enhancement of the number of potential applications.[^14^, ^15^]

Hierarchical porosity has attracted a growing body of work in porous materials.^[16]^ However, this interest has mainly been successful in coordinatively and covalently extended frameworks,^[17]^ where it has been possible to obtain single crystals with hierarchical porosity.^[18]^ For porous organic molecular materials, in contrast, the field is far behind.^[19]^ A prevailing reason may be the difficulty of controlling subtler interactions that direct the crystallisation of purely organic molecular materials. Furthermore, during the crystallisation, molecular materials tend to reduce any possible voids produced in the packing. Nonetheless, to integrate hierarchical micro‐ and macropores into a single crystal material requires overcoming the challenges derived from ligand size limitations and stability issues. Although some clever strategies have been proposed to integrate high order macroporosity into some of these molecular materials,^[6]^ the synthesis of single crystals of molecular materials with ordered micro‐ and macropores is yet to be achieved. Herein, we report the first example of a HOF‐based molecular material that can be obtained as single crystal with microporosity, termed as **M‐TPY** (where **M‐TPY**=microporous tripyridine), and hierarchical micro‐ and macroporosity, termed as **MM‐TPY** (where **MM‐TPY**=micro‐macroporous‐tripyridine). By controlling the rate of solvent evaporation, and the concentration of precursor during crystallisation, single crystals of different sizes, from μm to cm scale, can be obtained in a single step, without the use of any additives or templates. The assembly is mainly directed by the strong π‐π stacking interactions derived from the extended aromaticity in combination with the less common weaker, C−H⋅⋅⋅N bond interactions.^[20]^ The mechanism of assembly is based on the difference in energy strength between interactions, which favours skeletal growth during the solvent evaporation. As proof of concept for a novel application, we tested the hierarchical porosity as a platform for the selective co‐recognition of molecular species and microparticles.

## Results and Discussion

**TPY** was synthesized by adapting a reported procedure and characterised as described in the Supporting Information. **M‐TPY**,[C_3_N_3_{(C_6_H_4_)−(C_5_H_4_N)}_3_]⋅0.5 (CH_2_Cl_2_)⋅0.5 (EtOH) crystals with blade morphology were crystallised from a solution of **TPY** in dichloromethane/ethanol and structurally characterised by SCXRD (Table S1),^[21]^ revealing the porous nature of this material with dimensions of 9.07×7.50 Å, given the roughly rectangular cross‐section, so that gives 68 Å^2^, where the longest diagonal distance within each pore is 1.2 nm, thus confirming the nanometric porosity. The Platon Squeeze procedure^[22]^ was used with a probe size of 1.20 Å, which recovered 299 electrons over four voids representing a total of ca. 17 % of void space for the unit cell volume, and each with a volume of 266 Å^3^; thus ca. 75 electrons per void. This was interpreted as one solvent molecule of each type in each void, or half a molecule of each per **TPY** molecule.

**Figure 1:**
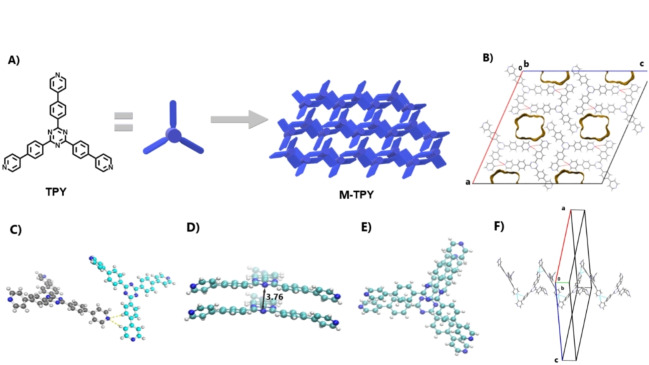
A) Chemical structure of precursor **TPY** and representation of the crystal packing of **M‐TPY**. B) Crystal packing obtained from single crystal X‐ray data featuring the porosity. C)–F) Representations and different views of the molecular interactions in the packing arrangement for the weakly hydrogen bonded motif (C) and strong, slightly slipped π‐π stacking (D), (E). F) Packing orientation of **M‐TPY** relative to the unit cell axis.

The **TPY** molecule is not flat, with modest or substantial twist angles between bonded aromatic rings along the three arms of the molecule. Between the central C_3_N_3_ ring and the middle C_6_H_4_ rings, the twist angles are quite shallow at between 5.51(16) and 9.61(15)°. The twist angles between the middle C_6_H_4_ rings and the outer pyridyl rings are much larger at between 28.70(12) and 38.71(10)° (Figure 1C). Despite this, all the aromatic groups of the **TPY** molecule take part in the formation of slightly slipped π‐π stacking interactions in solid state (Figure 1D,E). This occurs via simple translation along the *b* direction and with a small off‐set angle of *ca*. 12°. The shortest π‐π C⋅⋅⋅C contacts are in the range ca. 3.41–3.53 Å, while the shortest C⋅⋅⋅N contacts are somewhat shorter at *ca*. 3.37–3.46 Å. These significantly strong π‐π stacking interactions contribute to the stability of the porous framework. Molecules form spirals in the *b* direction and non‐symmetric layers in the a/c plane whereby spirals are linked together (Figure 1B, F, Figure S4b). The spirals are generated via 1 : 1 bifurcated interactions between N(4) and both H(19) and H(25) on the adjacent molecule with distances of 2.73 and 2.74 Å, respectively (Figure S4a). A layer is generated via interactions of N(5) with atom H(30) in a molecule above and H(36) in a different molecule below, both with distances of 2.76 Å. The pores formed in the structure form infinite channels, also along *b*. To measure the contribution of each interaction, density functional theory (DFT) calculations were performed on dimers, trimers, and tetramers of TPY as found within the small crystal structure (Figure S5, Table S2). These computations reveal a substantial π‐π stacking energy of ≈36 kcal mol^−1^ for the structure shown in Figure 1D. The energies for H‐bonds (5.4 kcal mol^−1^, Figure 1C) and side‐on interlayer interactions (4.5 kcal mol^−1^, Figure S4B) are considerably weaker. The presence of strong stacking interactions is also consistent with an interplane stacking distance of only 3.36 Å between dimers in the crystal packing, strongly supporting that the structural stability is mainly attributed to the extended aromatic framework. A similar trend in values was also confirmed by using CrystalExplorer (Table S3).^[23]^ The bulk material and its phase, measured by powder X‐ray diffraction (PXRD), retains the crystalline phase of the simulated PXRD pattern from the single crystal study (Figure S20). The thermal stability of the **TPY** building block was studied by thermogravimetric analysis (TGA) under N_2_ and air. The TGA curve under N_2_ (Figure S7) experienced an initial and gradual 12 % weight loss below 90 °C, attributed to the loss of solvent within the pores. A second weight loss of >50 % between 420 and 550 °C was attributed to thermal decomposition. This confirms that the **TPY** building block is thermally stable up to 400 °C. Similar results were confirmed under air (Figure S8) which further confirms a high thermal decomposition temperature. The **M‐TPY** framework thermal stability was tested by VT‐PXRD (Figure S9). This analysis confirmed that the framework remains stable up to 200 °C and above this temperature, changes in the framework are present, with the possible formation of a new phase.

**Figure 2:**
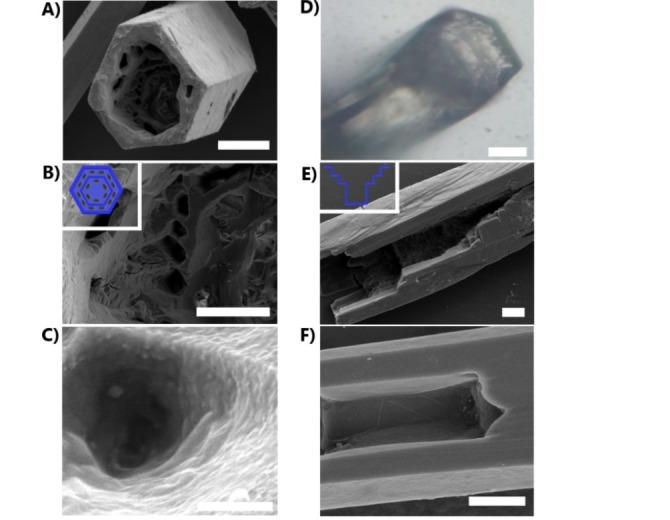
A) SEM image of the semi‐hollow tubular morphology of a regular μm size crystal of **MM‐TPY** obtained during fast crystallisation. B) In the interior of the macropore can be seen an array of minor micropores C) Closer look inside one of the macropores D) Optical microscopy image of one of the crystals featuring the layered staircase morphology. E) The staircase morphology is visible from a fragmented crystal and F) Bottom of the staircase morphology defining semi‐hollow tubules. Scales: A), D) 100 μm. B), E) 10 μm. C) 1 μm and F) 5 μm.

**Figure 3:**
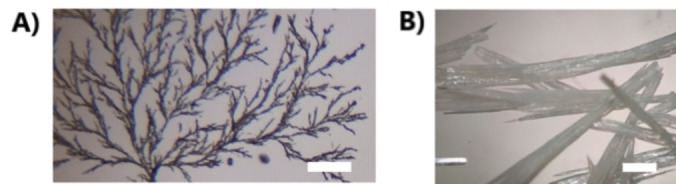
A) Optical image of dendritic morphology formed by increasing the concentration of **TPY** solvent evaporation, typical of skeletal growth. Scale bar: 100 μm. B) Image of large **MM‐TPY** crystals obtained after 3 days of crystallisation during a low evaporation rate of toluene. Scale bar: 1.5 mm.

**Figure 4:**
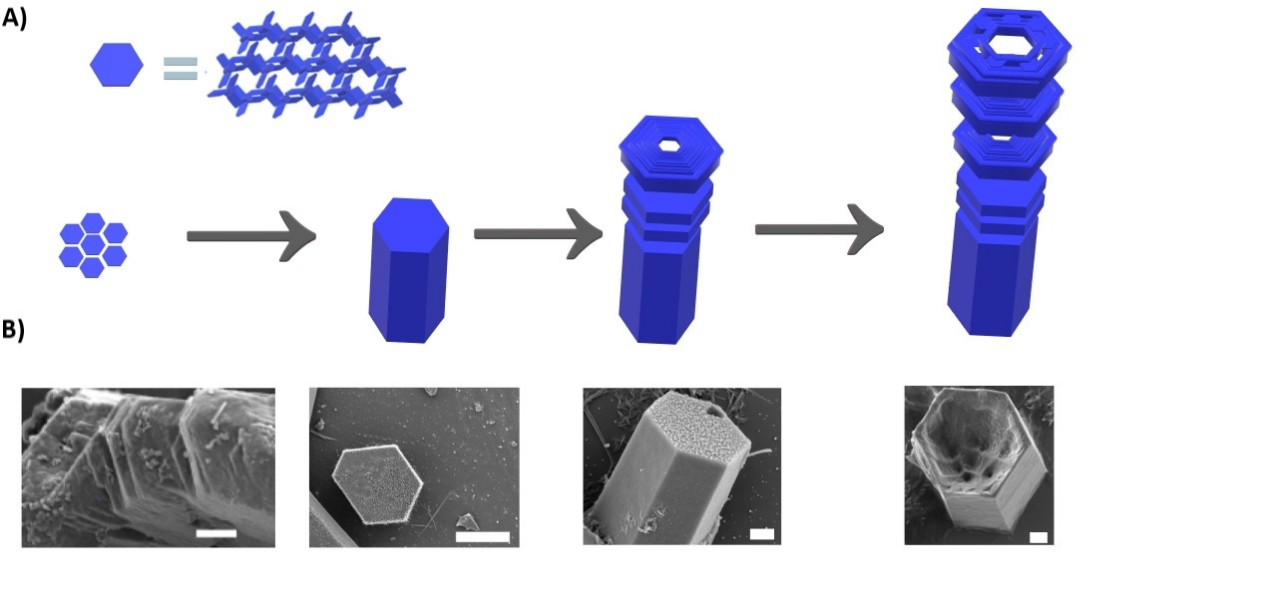
A) Top: representation of the platelets as hexagonal blue blocks. Bottom: representation of the proposed mechanism of growth for **MM‐TPY** crystals with tubular hierarchical morphology. B) SEM images of crystals in each stage of the crystal growth. Scale bars (in order, from left to right): 1 μm, 10 μm, 2 μm, 2 μm.

The porosity of **M‐TPY** was also studied by gas adsorption isotherms after sample activation (Figure S10, S11). The CO_2_ adsorption isotherm at 273 and 223 K of **M‐TPY** shows an uptake capacity of 1.33 and 1.82 mmol g^−1^, respectively, and BET surface area of 192 m^2^ g^−1^ (Figure S10). Meanwhile, N_2_ adsorption at 77 K was 0.88 mmol g^−1^ with a calculated median pore size of 6.9 Å. (Figure S11). This suggests a selective CO_2_ vs N_2_ adsorption, which most likely is attributed to the special quadrupole moment of CO_2,_ that would increase the framework interaction with CO_2_ molecules, compared with N_2_.^[24]^ The adsorption capacity and gas selectivity for **M‐TPY** are in the same range as other porous organic molecular materials with moderate adsorption.^[25]^ By modifying the crystallisation conditions of **TPY** by using toluene as solvent, and altering the rate of evaporation, we can introduce additional macroporosity (Figure 2) in a hierarchical manner and the size of the **MM‐TPY** crystals formed can be controlled, from μm size to cm (Figure 3). Initial examination by optical microscopy of the vertically orientated crystals, obtained by fast evaporation, reveals that the crystals assemble predominantly as semi‐hollow tubes, defining a main macropore. Scanning electron microscopy (SEM) also confirmed that **MM‐TPY** grows as crystals with a hexagonal cross section and tubular morphology (Figure 2). Further examination showed that the interior of the main macropore is characterised by a hierarchical structure that resembles a staircase wall morphology^[26]^ (Figure 2A,E, S12). A closer inspection of the interior by SEM indicated the presence of smaller macropores with diameters of the range of μm on each level of the staircase structure. The macroporosity was also studied by mercury intrusion porosimetry (Figure S13). Crystals with a main macropore centered at 173 μm show a hierarchical macroporosity centered at 72 and 3.5 μm. The presence of macropores within the major macropore represents another level of hierarchical porosity. SEM analysis of fragmented crystals confirmed that all macropores are accessible and the absence of additional hidden macropores (Figure S14). This was also corroborated by analysis using Focused Ion Beam Scanning Electron Microscopy (FIB‐SEM) of cross‐sectional areas (Figure S14). This type of crystal with such a complex hierarchical microporosity with different levels of macroporosity is unique for a template‐free assembled porous material. The CO_2_ adsorption isotherm of **MM‐TPY** is 0.7 mmol g^−1^ at 273 K and 1.7 mmol g^−1^ at 223 K, which represents a calculated BET surface area of 172 m^2^ g^−1^ (Figure S16). This suggests that the reduced CO_2_ sorption capability was due to the presence of macropores. PXRD comparison of activated **MM‐TPY** with **M‐TPY** confirmed that the crystalline phase is retained, and its stability in different conditions, including water and some organic solvents like ethyl acetate, hexane and acetonitrile although the stability is compromised in dimethyl formamide (Figure S20, S21). With the aim of gaining insight into the crystal growth mechanism, the crystal growth process was monitored by SEM and optical microscopy during the evaporation of a drop of toluene solution on a glass surface. These crystals can grow vertically from a glass surface such as a glass slide. SEM analysis of **MM‐TPY** crystals suggests that the tubular structures grow by the piling‐up of small platelets (Figure 4B). This could indicate that the extended aromatic π‐system from **TPY** promotes a growth in this direction through one‐dimensional π‐π stacking interactions, the growth direction of the hexagonal crystals along the *b*‐axis or the longest crystal length. The fast‐vertical growth in this direction, forming the outer walls, differs with the slower and uneven growth in the directions that correspond to the orientation of the C−H⋅⋅⋅N and side‐on interlayer interactions, mainly due to their weaker strength compared with the π‐π interactions. Thus, the growth is preferentially as tubular formations with a semi‐hollow interior. We hypothesize that the formation of hierarchical structures is due to this highly anisotropic growth. As the crystals grow and the concentration of **TPY** is depleted within the solvent trapped, the anisotropic growth rate is accelerated, which results in a staircase shaped interior. This type of growth is termed skeletal crystal growth^[26]^ and is present in conditions of high supersaturation levels, favoured by the low solubility of **TPY** in toluene at room temperature, and diffusion‐limited conditions. Although this mechanism is more common in inorganic crystals than in organic crystals, a few reports can be found suggesting similar diffusion‐limited mechanism growth, promoting highly anisotropic growth.^[27]^ This hypothesis was tested by stirring a solution of **TPY** in toluene during the solvent evaporation, where only solid morphologies were obtained, which suggested that diffusion‐limited conditions are required for the formation of hierarchical macroporosity. Further confirmation of this type of growth would be the presence of dendritic morphology,^[26]^ which was also observed during the assembly of **MM‐TPY** when the concentration of **TPY** in toluene was increased above 2 mg mL^−1^ during crystallisation (Figure 3A). Another factor that affected the formation of tubular hierarchical structures in **MM‐TPY** was the presence of impurities. We attempted to obtain hollow crystals of **HOF‐TPY** from the **TPY** precursor in the presence of a small number of impurities produced during the reaction synthesis. However, only solid, non‐hollow structures were obtained, which further confirms the hypothesis that π‐π stacking could play a key role on the assembly of cavities (Figure S22).

**Figure 5:**
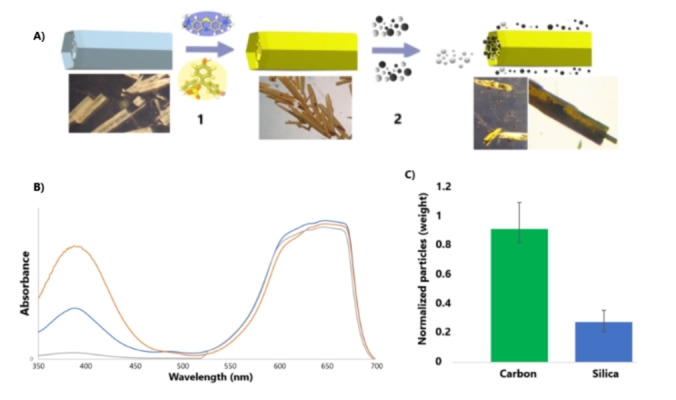
A) Top: Sequential and selective recognition of dyes and solid microparticles with **MM‐TPY** crystals, in a sequential process (**1** and **2**). **1**: The blue colored dye represents Methylene Blue, yellow colored dye represents Phenol Red. **2**: white spheres represent silica microparticles and black spheres represent carbon microparticles. Bottom: Optical microscopy images of the crystals during the process. B) Evolution of UV/Vis absorbance during the dye adsorption. C) Normalized graph for the total weight of carbon and silica particles attached to the crystals.

The formation of the smaller hierarchical macropores within the main tubular defined major macropore could be the result of the constant decrease in the concentration of **TPY** within the macropore during the fast crystal growth, and the difference in growth rate of the hydrogen bonding and side‐on interactions, leaving smaller macropores in each staircase level. **MM‐TPY** can be crystallised in a range of sizes, from μm to cm scale (Figure S23). By reducing the solvent evaporation rate, larger crystals with a more pronounced hollow interior, due to prolonged and accelerated anisotropic growth can be easily formed. Thus, we were able to obtain cm scale crystals of **MM‐TPY** (Figure 3B). The ability to grow cm scale crystals in porous molecular materials is unreported. Interestingly, this could have a diverse range of implications in different applications.^[28]^ In this respect, as proof of concept, we explored the use of the hierarchical porosity nature of **MM‐TPY** for the dual, sequential, and selective co‐recognition of molecular species and solid microparticles (Figure 5). Although the selective adsorption of molecular species in solution is well studied, the selective recognition of solids is less explored and very challenging.[^29^, ^30^] For this reason, first, we studied the adsorption of molecular species as dyes. Figure 5A indicates that **MM‐TPY** crystals experienced a change of colour to pale‐yellow when immersed in a mixture of dyes, Methylene Blue (MB) and Phenol Red (PR), in acetone, which suggests that PR is selectively adsorbed over MB. UV/Vis spectroscopy (Figure 5B) of the solution was used to follow the adsorption, showing a significant decrease in the band for PR with no change in intensity for the band of Methylene Blue. Desorption experiments further confirmed that the PR is selectively removed from the mixture (Figure S24). We have studied in more detail the preferential mechanism of adsorption of PR over MB. The molecular dimensions of both dyes, MB and PR, are 14.2×5.0×4.0 and 10.0×9.4×6.0 Å, respectively.^[31]^ The larger molecular dimension for MB (14.2 Å) compared with the longest pore dimensions, precludes in principle from its pore adsorption in some molecular orientations while the PR could be preferentially adsorbed.^[31a]^ Further analysis for the dye adsorption was done by FT‐IR (Figure S25). The IR spectra of **MM‐TPY** and PR@**MM‐TPY** show a shift (Δ*ṽ*≈10 cm^−1^) for the peaks that correspond to the triazine group of **MM‐TPY**, centered at 1362 and 1416 cm^−1^, which suggests an interaction between the N and the dye. This interaction could be a hydrogen bond between N of the triazine group and the OH groups in PR, which is also corroborated with the shift of the bands assigned to the OH groups in the PR (Δ*ṽ*≈60 cm^−1^). Then, in a sequential manner to the dye adsorption process, the crystals with the adsorbed dye (PR@**MM‐TPY**) were used for the recognition of particles within the macropores. A mixture of carbon and silica microparticles with a different range of μm sizes were used. The particles were mixed in a suspension of acetonitrile/water (9/1) and deposited on glass slides along with crystals of PR@**MM‐TPY** until the solvent evaporated. Optical microscopy images showed that mainly carbon particles are selectively attached on the outer surface and within the macropores. This was also confirmed after particle desorption, showing a ratio 7 : 1 of carbon and silica microparticles, respectively (Figure 5C). In order to rationalise the mechanism of adsorption within the macropores, the zeta potential (ξ) of **MM‐TPY** crystals and particles (Figure S26) was measured. As the data indicates, the surface of the PR@**MM‐TPY** crystals was negatively charged with a zeta potential of −0.36 mV. The measured zeta potential for the **MM‐TPY** crystals in the absence of adsorbed dye was 0.41 mV, which indicates that the ionic nature of the dye modifies the surface of the crystals. The measured zeta potentials for carbon and silica particles are −0.67 and −0.14 mV, respectively, which suggests that the preferential adsorption of carbon particles is not caused by electrostatic interactions on the crystal surface. A possible explanation of the preferential adsorption of carbon particles could be due to the π‐π stacking interactions between the extended aromatic framework forming the **MM‐TPY** crystals and the graphene layers from the activated carbon particles.^[32]^ The presented experiments are just a simple example of the potential of hierarchical porosity, which suggests that these materials with hierarchical nature and increased surface area to volume ratios could be used in future for different applications that require separation of solids and selective extraction of molecular species in solution, such as environmental recycling of materials,^[33]^ dual drug delivery of biological samples,^[34]^ and dual recycling of graphite and dyes.^[35]^

## Conclusion

Porous organic molecular materials are an emergent field with a unique combination of properties. However, the field is in its infancy when it comes to the introduction of hierarchical porosity in these materials. Herein, we have reported the first porous organic molecular material (**MM‐TPY**) based on a HOF that can crystallise with complex hierarchical micro‐and macroporous structure by pure self‐assembly. Crystals of different size scale, from μm to cm scale can be easily obtained in a single step without the addition of any template, surfactants, or modulators. The mechanism of assembly and crystal formation was investigated, and it is compatible with a highly anisotropic crystal growth due to the difference in energy among the highly orientated π‐π, C−H⋅⋅⋅N and side‐on interactions during diffusion‐limited and supersaturated conditions. We proposed that choosing a solvent that favours skeletal growth during crystallisation, in combination with a better control of the balance between hydrogen bonds and π‐π interactions, could be key to obtain complex hierarchical porosity in more molecular materials purely by self‐assembly. The formation of hierarchical porous molecular materials with complex architectures and at different scales, from μm to cm, is a milestone that could lead to new properties and therefore novel applications. As proof of concept, the use of hierarchical porosity for the dual and selective co‐recognition of molecules and microparticles was introduced, which could be relevant for several applications.

## Experimental Section

Essential Experimental Procedures/Data.

## Conflict of interest

The authors declare no conflict of interest.

1

## Supporting information

As a service to our authors and readers, this journal provides supporting information supplied by the authors. Such materials are peer reviewed and may be re‐organized for online delivery, but are not copy‐edited or typeset. Technical support issues arising from supporting information (other than missing files) should be addressed to the authors.

Supporting Information

## Data Availability

The data that support the findings of this study are available from the corresponding author upon reasonable request.

## References

[1a] A. I. Cooper , ACS Cent. Sci. 2017, 3, 544–553; 28691065 10.1021/acscentsci.7b00146 PMC5492258

[1b] M. A. Little , A. I. Cooper , Adv. Funct. Mater. 2020, 30, 1909842.

[2] S. Feng , Y. Shang , Z. Wang , Z. Kang , R. Wang , J. Jiang , L. Fan , W. Fan , Z. Liu , G. Kong , Y. Feng , S. Hu , H. Guo , D. Sun , Angew. Chem. Int. Ed. 2020, 59, 3840–3845; 10.1002/anie.201914548 31833627

[3] Q. Wang , D. Astruc , Chem. Rev. 2020, 120, 1438–1511. 31246430 10.1021/acs.chemrev.9b00223

[4] K. Geng , T. He , R. Liu , S. Dalapati , K. T. Tan , Z. Li , S. Tao , Y. Gong , Q. Jiang , D. Jiang , Chem. Rev. 2020, 120, 8814–8933. 31967791 10.1021/acs.chemrev.9b00550

[5] Z. Tang , X. Li , L. Tong , H. Yang , J. Wu , X. Zhang , T. Song , S. Huang , F. Zhu , G. Chen , G. Ouyang , Angew. Chem. Int. Ed. 2021, 60, 23608–23613; 10.1002/anie.202110351 34459532

[6] S. Yu , G.-L. Xing , L.-H. Chen , T. Ben , B.-L. Su , Adv. Mater. 2020, 32, 2003270. 10.1002/adma.202003270 32930443

[7] T. Hasell , A. I. Cooper , Nat. Rev. Mater. 2016, 1, 16053.

[8] Y. Tian , J. Yang , M. Gao , Z. Liu , M. Zhao , M. Fang , Z. Li , Mater. Horiz. 2022, 9, 731–739. 34859253 10.1039/d1mh01360b

[9] D. Meng , J. L. Yang , C. Xiao , R. Wang , X. Xing , O. Kocak , G. Aydin , I. Yavuz , S. Nuryyeva , L. Zhang , G. Liu , Z. Li , S. Yuan , Z.-K. Wang , W. Wei , Z. Wang , K. N. Houk , Y. Yang , Proc. Natl. Acad. Sci. USA 2020, 117, 20397–20403. 32788358 10.1073/pnas.2010733117 PMC7456091

[10] J. Tian , H. Wang , D.-W. Zhang , Y. Liu , Z.-T. Li , Natl. Sci. Rev. 2017, 4, 426–436.

[11] G. Gong , S. Lv , J. Han , F. Xie , Q. Li , N. Xia , W. Zeng , Y. Chen , L. Wang , J. Wang , S. Chen , Angew. Chem. Int. Ed. 2021, 60, 14831–14835; 10.1002/anie.202102448 33872474

[12] G. Zhang , B. Hua , A. Dey , M. Ghosh , B. A. Moosa , N. M. Khashab , Acc. Chem. Res. 2021, 54, 155–168. 33332097 10.1021/acs.accounts.0c00582

[13] I. Hisaki , C. Xin , K. Takahashi , T. Nakamura , Angew. Chem. Int. Ed. 2019, 58, 11160–11170; 10.1002/anie.201902147 30891889

[14a] H. V. Doan , H. A. Hamzah , P. K. Prabhakaran , C. Petrillo , V. P. Ting , Nano-Micro Lett. 2019, 11, 54; 10.1007/s40820-019-0286-9 PMC7770918 34137991

[14b] L. Feng , K.-Y. Wang , J. Willman , H.-C. Zhou , ACS Cent. Sci. 2020, 6, 359–367. 32232136 10.1021/acscentsci.0c00158 PMC7099594

[15] W. Schwieger , A. G. Machoke , T. Weissenberger , A. Inayat , T. Selvam , M. Klumpp , A. Inayat , Chem. Soc. Rev. 2016, 45, 3353–3376. 26477329 10.1039/c5cs00599j

[16a] L. Feng , K.-Y. Wang , X.-L. Lv , T.-H. Yan , H.-C. Zhou , Natl. Sci. Rev. 2020, 7, 1743–1758; 34691505 10.1093/nsr/nwz170 PMC8290954

[16b] L. Feng , K.-Y. Wang , J. Powell , H.-C. Zhou , Matter 2019, 1, 801–824.

[17a] X. Zhao , P. Pachfule , S. Li , T. Langenham , M. Ye , X. Sclesiger , S. Paetz , J. Schmidt , A. Thomas , J. Am. Chem. Soc. 2019, 141, 6623–6630; 30916950 10.1021/jacs.9b01226

[17b] R.-R. Liang , S.-Y. Jiang , R.-H. A , X. Zhao , Chem. Soc. Rev. 2020, 49, 3920. 32427238 10.1039/d0cs00049c

[18] K. Shen , L. Zhang , X. Chen , L. Liu , D. Zhang , Y. Han , J. Chen , J. Long , R. Luque , Y. Li , B. Chen , Science 2018, 359, 206–210. 29326271 10.1126/science.aao3403

[19a] Q. Yin , Y.-L. Li , L. Li , J. Lü , T.-F. Liu , R. Cao , ACS Appl. Mater. Interfaces 2019, 11, 17823–17827; 31009575 10.1021/acsami.9b03696

[19b] L. K. Shrestha , Y. Yamauchi , J. P. Hill , K. Miyazawa , K. Ariga , J. Am. Chem. Soc. 2013, 135, 586–589; 23276230 10.1021/ja3108752

[19c] M. Hua , S. Wang , Y. Gong , J. Wei , Z. Yang , J.-K. Sun , Angew. Chem. Int. Ed. 2021, 60, 12490–12497; 10.1002/anie.202100849 33694301

[20] H. Yamagishi , H. Sato , A. Hori , Y. Sator , O. Matsuda , K. Kato , T. Aida , Science 2018, 361, 1242–1246. 30237354 10.1126/science.aat6394

[21] Deposition Number 2164359 contains the supplementary crystallographic data for this paper. These data are provided free of charge by the joint Cambridge Crystallographic Data Centre and Fachinformationszentrum Karlsruhe Access Structures service.

[22a] P. van der Sluis , A. L. Spek , Acta Crystallogr. Sect. A 1990, 46, 194–201;

[22b] A. L. Spek , Acta Crystallogr. Sect. C 2015, 71, 9–18. 10.1107/S2053229614024929 25567569

[23] P. R. Spackman , M. J. Turner , J. J. McKinnon , S. K. Wolff , D. J. Grimwood , D. Jayatilaka , M. A. Spackman , J. Appl. Crystallogr. 2021, 54, 1006–1011. 34188619 10.1107/S1600576721002910 PMC8202033

[24] P. Wei , X. He , Z. Zheng , D. He , Q. Li , J. Gong , J. Zhang , H. H. Y. Sung , I. D. Williams , J. W. Y. Lam , M. Liu , B. Z. Tang , Angew. Chem. Int. Ed. 2021, 60, 7148–7154; 10.1002/anie.202013117 33300645

[25a] B.-T. Liu , X.-H. Pan , D.-Y. Zhang , R. Wang , J.-Y. Chen , H.-R. Fang , T.-F. Liu , Angew. Chem. Int. Ed. 2021, 60, 25701–25707; 10.1002/anie.202110028 34477299

[25b] Q. Huang , W. Li , Z. Mao , L. Qu , Y. Li , H. Zhang , T. Yu , Z. Yang , J. Zhao , Y. Zhang , M. P. Aldred , Z. Chi , Nat. Commun. 2019, 10, 3074. 31300644 10.1038/s41467-019-10575-5 PMC6625987

[26] Z. Yang , J. Zhang , L. Zhang , B. Fu , P. Tao , C. Song , W. Shang , T. Deng , Adv. Funct. Mater. 2020, 30, 1908108.

[27] M. Li , C. Zhang , M. Li , F. Liu , L. Zhou , Z. Gao , J. Sun , D. Han , J. Gong , Chem. Eng. J. 2022, 429, 132450.

[28a] A. G. Zavyalova , D. V. Kladko , I. Y. Chernyshov , V. V. Vinogradov , J. Mater. Chem. A 2021, 9, 25258–25271;

[28b] P. Falcaro , K. Okada , T. Hara , K. Ikigaki , Y. Tokudome , A. W. Thornton , A. J. Hill , T. Williams , C. Doonan , M. Takahashi , Nat. Mater. 2017, 16, 342–348. 27918565 10.1038/nmat4815

[29a] Q. Tang , S. Maji , B. Jiang , J. Sun , W. Zhao , J. P. Hill , K. Ariga , H. Fuchs , Q. Ji , L. K. Shrestha , ACS Nano 2019, 13, 14005–14012; 31794176 10.1021/acsnano.9b05938

[29b] P. Bairi , K. Minami , J. P. Hill , K. Ariga , L. K. Shrestha , ACS Nano 2017, 11, 7790–7796. 28742325 10.1021/acsnano.7b01569

[30] X.-Y. Liu , F. Zhang , T.-W. Goh , Y. Li , Y.-C. Shao , L. Luo , W. Huang , Y.-T. Long , L.-Y. Chou , C.-K. Tsung , Angew. Chem. Int. Ed. 2018, 57, 2110–2114; 10.1002/anie.201711600 29266678

[31a] X. Xie , X. Huang , W. Lin , Y. Chen , X. Lang , Y. Wang , L. Gao , H. Zhu , J. Chen , ACS Omega 2020, 5, 13595–13600; 32566824 10.1021/acsomega.0c00385 PMC7301382

[31b] C. Huang , Z. Guo , X. Zhen , X. Chen , Z. Xue , S. Zhang , X. Li , B. Guan , X. Li , G. Hu , T. Wang , J. Am. Chem. Soc. 2020, 142, 9408–9414. 32302117 10.1021/jacs.0c02272

[32] S. Gil-Guerrero , N. Otero , M. Queizán , M. M. Alonso , Sensors 2019, 19, 1896. 31010075 10.3390/s19081896 PMC6514874

[33] N. Cao , Y. Zhang , L. Chen , W. Chu , Y. Huang , Y. Jia , M. Wang , J. Power Sources 2021, 483, 229163.

[34] T. Salafi , K. K. Zeming , Y. Zhang , Lab Chip 2017, 17, 11–33. 10.1039/c6lc01045h 27830852

[35] F. Schoden , M. Dotter , D. Knefelkamp , T. Blachowicz , E. S. Hellkamp , Energies 2021, 14, 3741.

