# Supplementary material for: Hierarchical Assembly of a Micro‐ and Macroporous Hydrogen‐Bonded Organic Framework with Tailored Single‐Crystal Size
Source: Angew Chem Int Ed Engl. 2022 Oct 19;61(47):e202208677. doi: 10.1002/anie.202208677 (PMC9827975; doi:10.1002/anie.202208677)
Supplement: Supplementary file 1 — Supporting Information [file ANIE-61-0-s001.pdf]

Angewandte Chemie
Eine Zeitschrift der Gesellschaft Deutscher Chemiker

Supporting Information

Hierarchical Assembly of a Micro- and Macroporous Hydrogen-Bonded Organic Framework with Tailored Single-Crystal Size

*C. A. Halliwell, S. E. Dann, J. Ferrando-Soria, F. Plasser, K. Yendall, E. V. Ramos-Fernandez, G. T. Vladisavljević, M. R. J. Elsegood, A. Fernandez**

General Materials and Analysis

Reactants, solvents, and other reagents were all commercially purchased from various suppliers and in all cases, were obtained in 95-100% purity. All chemicals were used directly without further purification, unless stated otherwise. Analytical thin-layer chromatography was used in most syntheses and were performed on 40 mm x 80 mm precoated Machery-Nagel ALUGRAM SIL G/UV_{254} aluminium sheets (Fischer Scientific) and visualised with ultraviolet light (264 nm). Column chromatography was performed using silica gel 60, 40-63 µm mesh (Apollo Scientific). ^{1}H NMR spectra were recorded on either a JEOL-ECS-400 FT (400 MHz) or a Jeol-ECZ-R-500 (500 MHz) spectrometer. ^{13}C NMR spectra were recorded on either a JEOL-ECS-400 FT (400 MHz) or a JEOL-ECZ-R-500 (500 MHz) spectrometer. Chemical shifts (δ) are reported in ppm downfield of $Si(CH_3)_4$, which was used as an internal standard. Coupling constants (*J*) are quoted in hertz and multiplicities are reported as singlets (s), doublets (d), triplets (t), doublet of doublets (dd), doublet of triplets (dt) or multiplets (m). NMR samples were measured at 298 K in solutions of $CDCl_3$. All NMR spectra were processed using the Delta software. Thermogravimetric analysis was conducted using a Mettler-Toledo model TGA/SDTA851 in a temperature range from 25 °C to 800 °C at a heating rate of 10 °C min^{-1} under N_2 and air with a flow rate of 5 ^{o}C min^{-1}. Single-crystal X-ray crystallography data was obtained from the ESPRC National Crystallographic Service at the University of Southampton and collected on a Rigaku 007FH diffractometer equipped with an AFC11 goniometer and HyPix 6000 detector at 100(2) K using Cu-Kα (λ = 1.54178 Å) radiation. The structure was solved by dual-space iterative methods and refined on F^2 by full-matrix least squares methods using the SHELXL-2018 software package. H atoms were constrained in a riding model. Refined as a 2-component twin with twin law 0.087 0 –0.913, 0 –1 0, –1.087 0 –0.087 and twin component ratio of 0.0407:0.9593(19). The solvate molecules were treated as diffuse contribution to the overall scattering without specific atom positions by SQUEEZE/PLATON due to severe disorder of these solvate molecules in the lattice. [4] Figures were assembled using Mercury or XP. Further details are provided in table S1 and in the deposited cif file CCDC 2164359 contains the supplementary crystallographic data for this

paper. These data can be obtained free of charge from The Cambridge Crystallographic Data Centre via www.ccdc.cam.ac.uk/structures. PXRD patterns were obtained using a Bruker D2 Phaser X-ray diffractometer, fitted with a 1-dimensional Lynxeye detector and a copper X-ray source (Kα = 1.54184 Å) operated at 30 kV and 10 mA. Kβ radiation was suppressed by means of a 0.5 mm thick nickel filter. Patterns were recorded over a range of 4 – 30° (2θ) with a step size of 0.041° and an equivalent step time of 96 seconds per step. Sample rotation was set at 15 rpm. Scanning electron microscopy was carried out on a JEOL JSM-7100F Field Emission Scanning Electron Microscope (FE-SEM). Samples were sputter coated with gold/palladium to provide a 5–10 nm conductive layer to facilitate analysis. Images were collected using an accelerating voltage of 5 kV and an approximate beam current of 120 pA. Zeta potential measurements were done in a Zetasizer nano ZS from Malvern Panalytical. Activated carbon and silica microparticles attached with a range of sizes from 2-60 μm to the crystals were analysed using previous published protocol [1]. UV-vis analysis was performed in a Shimadzu UV-1800 UV-Vis Spectrophotometer. N_2 and low T CO_2 gas adsorption analysis was performed in a Micromeritics ASAP 2020. High-pressure CO_2 gas adsorption measurements were performed in a homemade fully automated manometric equipment designed and constructed by the LMA group, now commercialized as iSorbHP by Quantachrome Instruments. CO_2 adsorption capacity was measured at 0°C and up to 2.5 MPa. All the samples were out-gases at 110°C for 12 hours prior the gas adsorption experiment.

6-Tris(4-(4-pyridinyl)phenyl]-1, 3, 5-triazine (TPY)

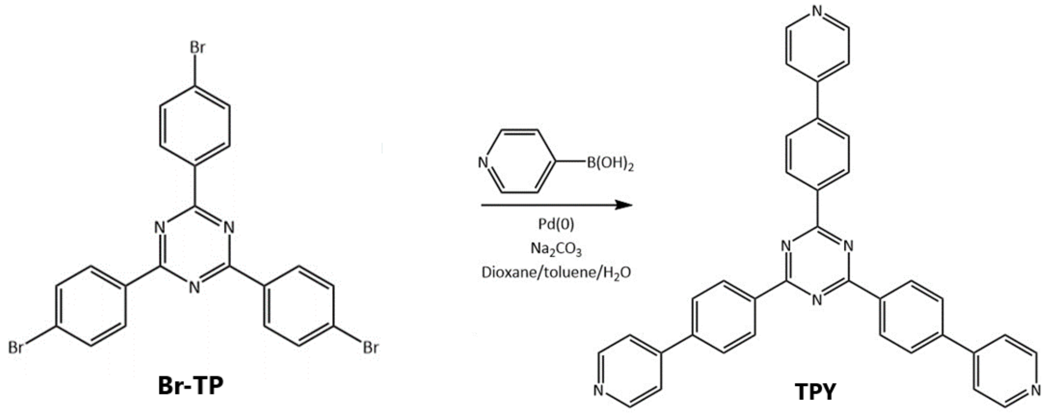

To a round-bottomed flask, **Br-TP** (1.0022 g, 3.15 mmol), 4-pyridinylboronic acid (3.0218 g, 24.58 mmol) and $Pd(PPh_3)_4$ (0.3658 g, 0.32 mmol) were suspended in 2:1 dioxane:toluene (60 mL) and degassed for 30 minutes. A solution of sodium carbonate (3.0208 g, 28.50 mmol) in distilled water (10 mL) was added to the mixture, which was then refluxed for 4 days under a nitrogen atmosphere. The mixture was cooled down, then the organic layer collected and washed with chloroform (3 x 30 mL) and distilled water (30 mL). Evaporating to dryness afforded **TPY** as off-white powder (1.54 g, 91%). ^{1}H-NMR (400 MHz, $CDCl_3$) δ 8.90 (dd, $^3J_{HH}$ 8 Hz, $^4J_{HH}$ 1.6 Hz, 6H, arom. H); 8.74 (dd, $^3J_{HH}$ 5.6 Hz, $^4J_{HH}$ 1.6 Hz, 6H, arom. H); 7.86 (dd, $^3J_{HH}$ 8.4 Hz, $^4J_{HH}$ 1.6 Hz, 6H, arom. H); 7.62 (dd, $^3J_{HH}$ 6Hz, $^4J_{HH}$ 1.6 Hz, 6H, arom. H) ppm. ^{13}C-NMR (400 MHz, $CDCl_3$) δ 171.3 (3C, arom. C); 150.6 (6C, arom. CH); 147.5 (3C, arom. C); 142.3 (3C, arom. C); 136.7 (3C, arom. C); 129.8 (6C, arom. CH); 127.4 (6C, arom. CH); 121.7 (6C, arom. CH) ppm.

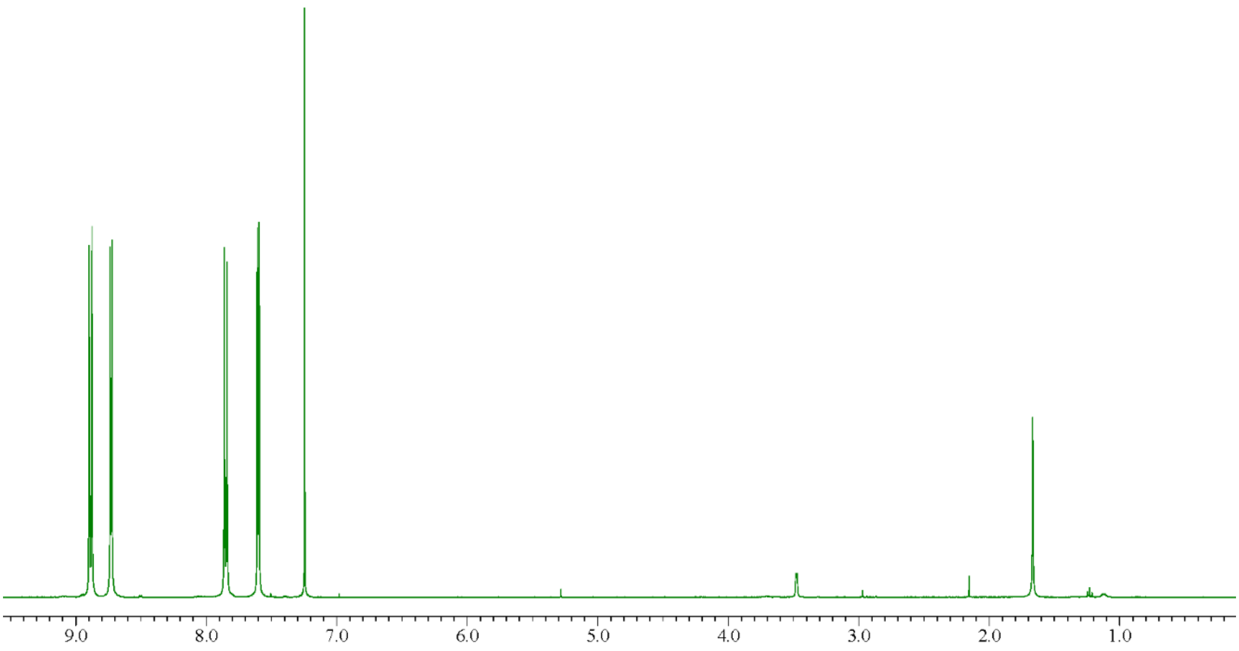

Figure S1. ^{1}H NMR spectrum of **TPY** in deuterated chloroform

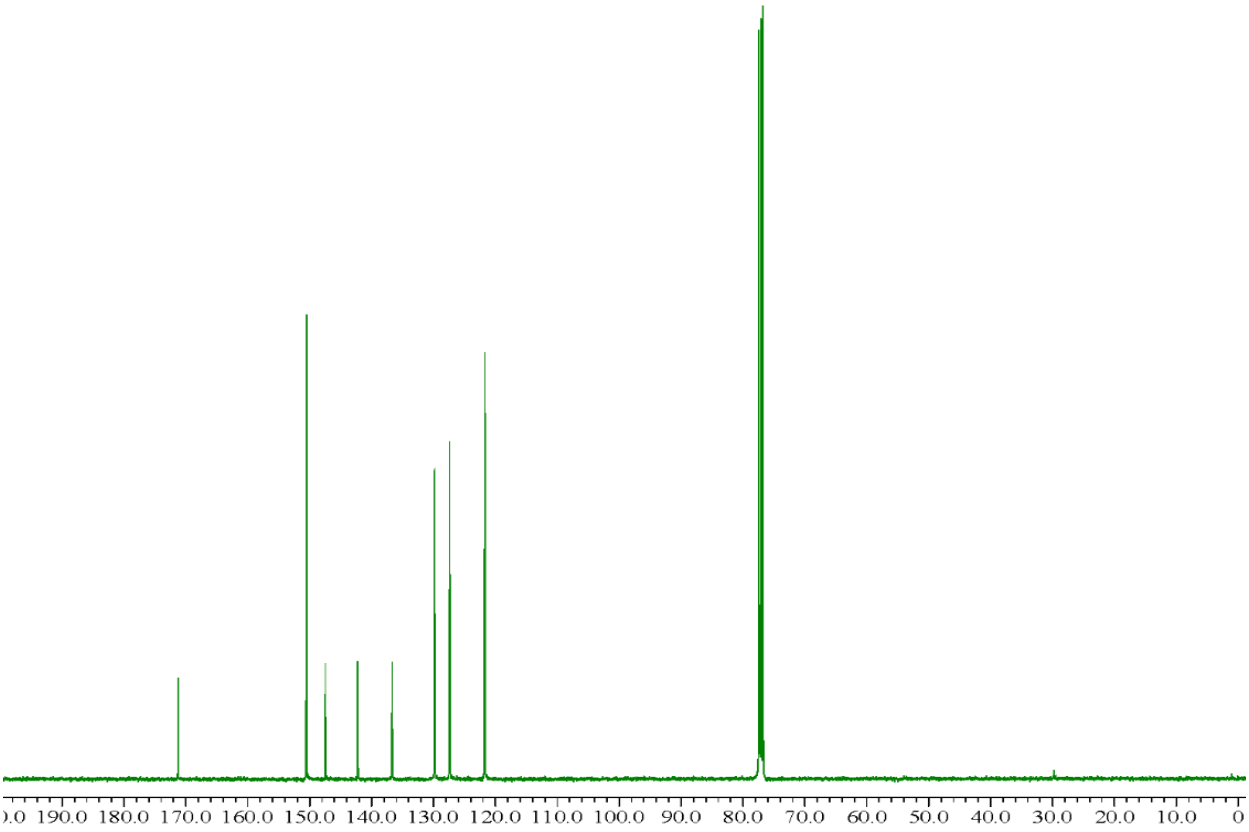

Figure S2. ^{13}C NMR spectrum of **TPY** in deuterated chloroform

M-TPY and MM-TPY formation and crystallisation:

-Dichloromethane/Ethanol (M-TPY): M-TPY was crystallised during a slow evaporation of a solution of **TPY** (10 mg) in dichloromethane, with the addition of ethanol after a few weeks. The obtained crystals are suitable for X-ray crystallography.

-Hierarchical tubular crystals (MM-TPY): TPY (10 mg) in toluene (15 mL) were added into a glass vial. The mixture was heated at 110° C for 10 minutes until complete dissolution. After all the toluene was evaporated at room temperature, the hollow crystals of **MM-TPY** were collected. By controlling the evaporation rate of toluene from hours to days, crystals of micrometre or mm scale can be obtained.

-Study of the mechanism of formation of hierarchical tubular crystals (MM-TPY): a few drops of a solution of 10 mg of **TPY** in toluene (15 mL) was deposited on a glass slide and left to evaporate quickly at room temperature (~20 minutes), resulting in micrometre scale crystals.

-Study for the selective co-recognition of dyes and microparticles: 1-Dye mixture: millimetre size crystals of **MM-TPY** were dipped in a mixture of Methylene Blue (MB) and Phenol Red (PR) in acetone for 48 h and then filtered, washed and dried for the next step. **2-Microparticle mixture:** a few drops of a suspension of activated carbon particles and silica gel particles in acetonitrile/water (9/1) were deposited on a glass slide containing crystals of PR@**MM-TPY** and left to complete evaporation. After the process was repeated 3 times, the crystals were gently washed with acetonitrile/water.

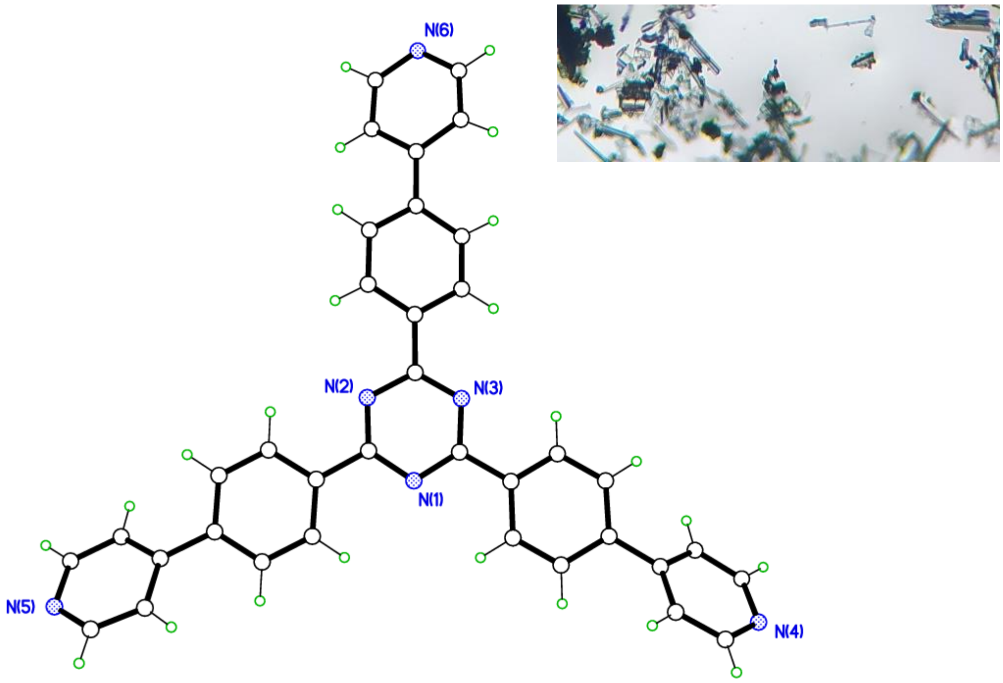

Figure S3. Crystal structure of **M-TPY**. Insert: image of the crystals obtained after crystallisation in a dichloromethane-ethanol mixture.

a) b)

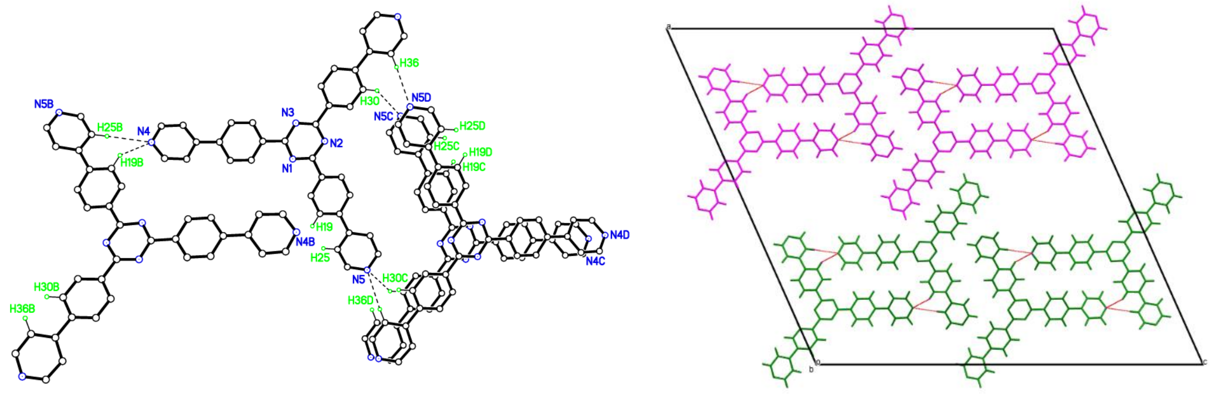

Figure S4. Overview of the crystal packing of **M-TPY**: a) detailed visualization of the hydrogen-bonding and b) visualization of the layered packing representing AB layers with H-bonding within layers and side-on interactions between layers

Table S1. Crystallographic data and structure refinement results of **M-TPY** crystallized in dichloromethane/ethanol mixture and face indexing.

1-Crystal data

$C_{36}H_{24}N_6{\cdot}0.5(C_2H_6O){\cdot}0.5(CH_2Cl_2)$	$F(000) = 2528$
$M_r = 606.11$	$D_x = 1.305$ Mg m^{-3}
Monoclinic, *C*2/*c*	Cu $K\alpha$ radiation, $\lambda = 1.54178$ Å
$a = 41.2612$ (12) Å	Cell parameters from 8673 reflections
$b = 3.76808$ (7) Å	$\theta = 3.8–69.8°$
$c = 43.4466$ (13) Å	$\mu = 1.40$ mm^{-1}
$\beta = 114.068$ (3)°	$T = 100$ K
$V = 6167.6$ (3) Å^3	Blade, colourless
$Z = 8$	$0.16 \times 0.04 \times 0.01$ mm^3

Experimental details

Crystal data	
Chemical formula	$C_{36}H_{24}N_6{\cdot}0.5(C_2H_6O){\cdot}0.5(CH_2Cl_2)$
M_r	606.11
Crystal system, space group	Monoclinic, *C*2/*c*
Temperature (K)	100
a, *b*, *c* (Å)	41.2612 (12), 3.76808 (7), 43.4466 (13)
β (°)	114.068 (3)

V (Å^3)	6167.6 (3)
Z	8
Radiation type	Cu $K\alpha$
μ (mm^{-1})	1.40
Crystal size (mm^3)	0.16 × 0.04 × 0.01
Data collection	
Diffractometer	Rigaku 007HF equipped with Varimax confocal mirrors and an AFC11 goniometer and HyPix 6000 detector.
Absorption correction	Empirical (using intensity measurements) *CrysAlis PRO* 1.171.40.79a (Rigaku Oxford Diffraction, 2020). Empirical absorption correction using spherical harmonics, implemented in SCALE3 ABSPACK scaling algorithm.
T_{min}, T_{max}	0.723, 1.000
No. of measured, independent and observed [$I > 2\sigma(I)$] reflections	40529, 5643, 4361
R_{int}	0.070
(sin θ/λ)$_{max}$ (Å^{-1})	0.602
Refinement	
$R[F^2 > 2\sigma(F^2)]$, $wR(F^2)$, S	0.090, 0.319, 1.08
No. of reflections	5643
No. of parameters	380
H-atom treatment	H-atom parameters constrained
	$w = 1/[\sigma^2(F_o^2) + (0.2046P)^2 + 13.190P]$ where $P = (F_o^2 + 2F_c^2)/3$
$\Delta\rangle_{max}$, $\Delta\rangle_{min}$ (e Å^{-3})	0.32, -0.36

2-Face indexing

h	k	l	Å
1	0	1	0.0798
-1	0	-1	0.1379
1	0	-1	0.0070
-1	0	1	0.0090
2	1	-2	0.0230
-2	-1	2	0.0184

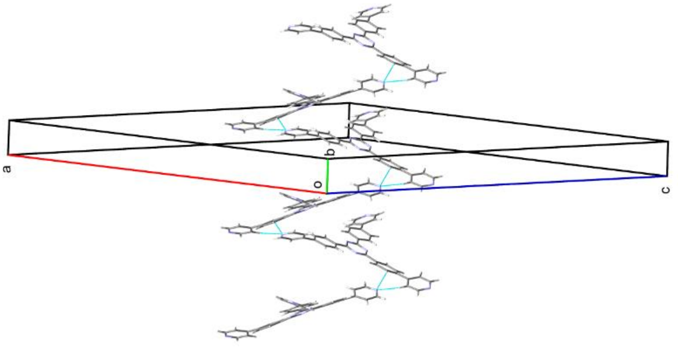

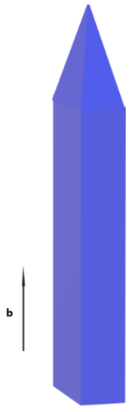

crystal orientation

Thus the 1 0 1 and –1 0 –1 planes are those at the top/bottom of the crystal when the long length is vertical i.e. separated by the longest distance. Hence the needle direction corresponds to the direction of growth of the spirals along *b*.

Computing details

Data collection: *CrysAlis PRO* 1.171.40.79a (Rigaku OD, 2020); cell refinement: *CrysAlis PRO* 1.171.40.79a (Rigaku OD, 2020); data reduction: *CrysAlis PRO* 1.171.40.79a (Rigaku OD, 2020); program(s) used to solve structure: *SHELXS97* (Sheldrick 2008); program(s) used to refine structure: *SHELXL2018*/3 (Sheldrick, 2018); molecular graphics: Bruker *SHELXTL*; software used to prepare material for publication: Bruker *SHELXTL*.

Density functional theory (DFT)

Density functional theory (DFT) computations were performed at two different levels of theory M06-2X/6-31G(d,p) and wB97M-V/def2-TZVP [2-5]. The binding energies presented below were obtained from single-point computations on monomers and dimers extracted from the crystal structure, without further structure optimization. All DFT computations were performed in Q-Chem 5.4 [6].

Computations were performed on six different structures to investigate the different types of interactions (Figure S5). A stacked dimer, trimer, and tetramer were considered. In addition, a dimer containing a hydrogen bond (N...H-C) and a dimer showing side-on interlayer interactions were considered. Finally, a mixed tetramer containing all types of interactions was used. Computations indicate that the stacking energies are between 34 and 37.5 kcal/mol per dimer interaction. These numbers are robust with respect to changes in the model size and computational model. H-bonded interactions are significantly smaller (5.39 and 4.13 kcal/mol for M06-2X and wB97M-V); and side-on interactions are slightly smaller than these (4.54 and 3.02 kcal/mol for M06-2X and wB97M-V). Finally, the mixed dimer has an overall binding energy of 87.67 kcal/mol, which is consistent with its constituent interactions (2 x stacking, 2 x H-bond, 1 x side-on) adding up to 87.36 kcal/mol.

Stacked dimer

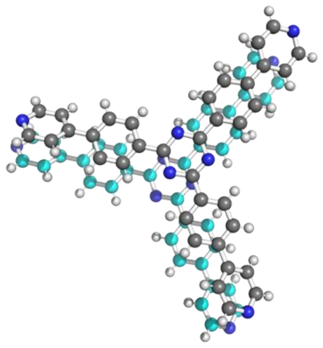

H-bonded dimer

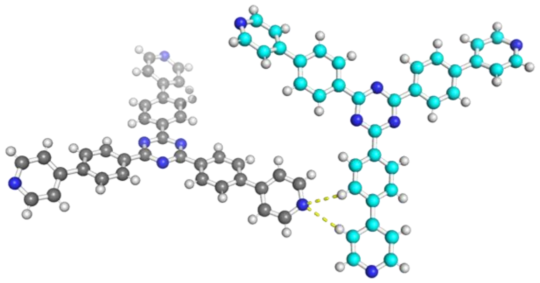

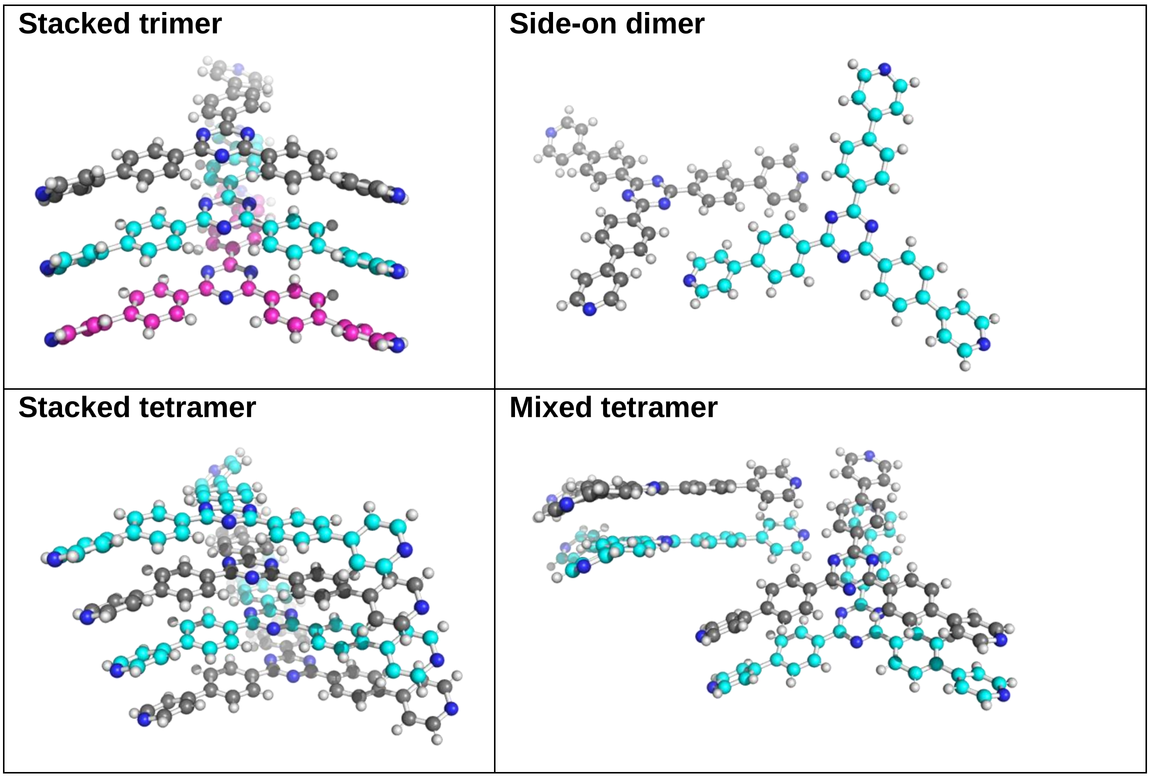

Figure S5. Dimer, trimer, and tetramer models used for the DFT computations.

Table S2. Computed binding energies (kcal/mol) of the structures presented in Figure S5 using two different density functionals. E_b represents the overall interaction energy of the multimer, E_b' represents the binding energy per dimer.

	E_b (M06-2X)	E_b' (M06-2X)	E_b (wB97M-V)	E_b' (wB97M-V)
Stacked dimer	36.02	36.02	34.06	34.06
Stacked trimer	73.45	36.73	70.17	35.08
Stacked tetramer	112.21	37.40		
H-bonded dimer	5.39	5.39	4.13	4.13
Side-on dimer	4.54	4.54	3.02	3.02
Mixed tetramer	87.67			

Table S3. Computed binding energies (kJ/mol) using CrystalExplorer for the 3 types of interactions in the crystal packing

	Electron Density	E_ele (kJ/mol)	E_pol (kJ/mol)	E_dis (kJ/mol)	E_rep (kJ/mol)	E_tot (kJ/mol)
π-π stacking	B3LYP/6-31G(d,p)	-7.2	-2.8	-198.6	114.3	-112.0
N...H-C (H-bond)	B3LYP/6-31G(d,p)	-2.9	-4.6	-39.3	0.0	-40.7

side-on interlayer interactions	B3LYP/6-31G(d,p)	-5.0	-2.9	-32.3	0.0	-35.6

E_tot represents the calculated total energy

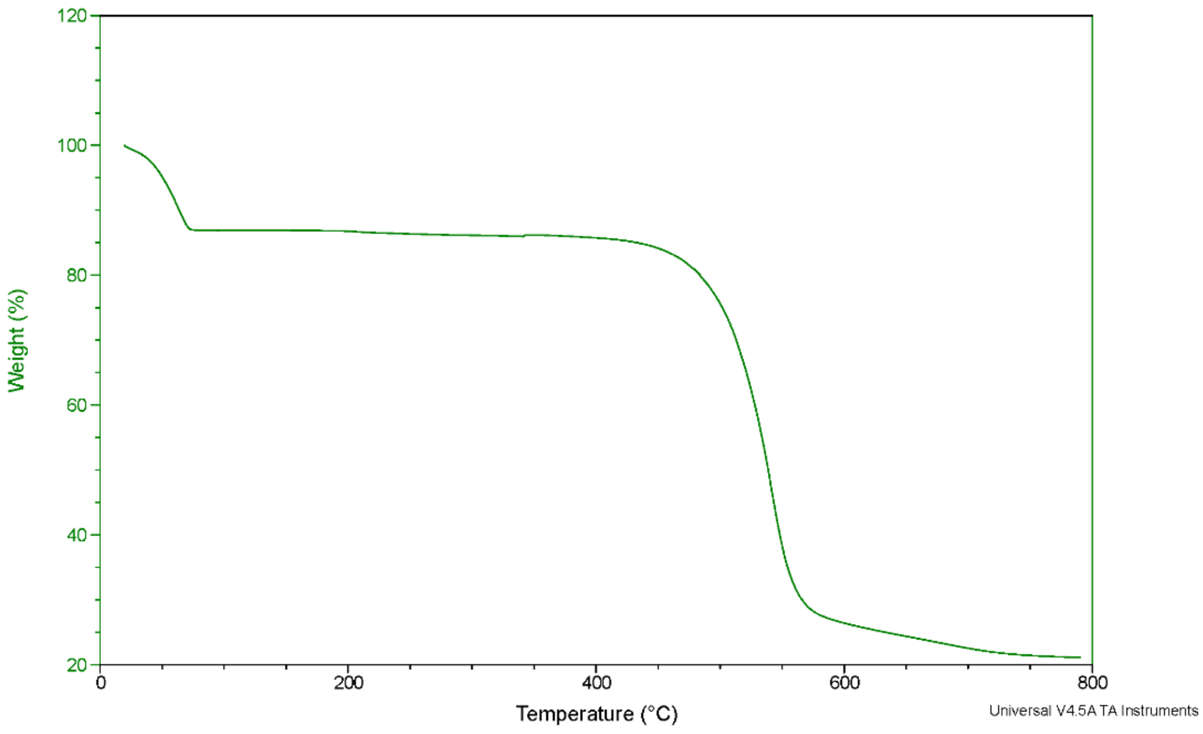

Figure S7. TGA curve under N_2 for the as-synthesized sample **M-TPY** crystallised in dichloromethane/ethanol, indicating a weight loss before 90 ^oC due to the guest solvent removal.

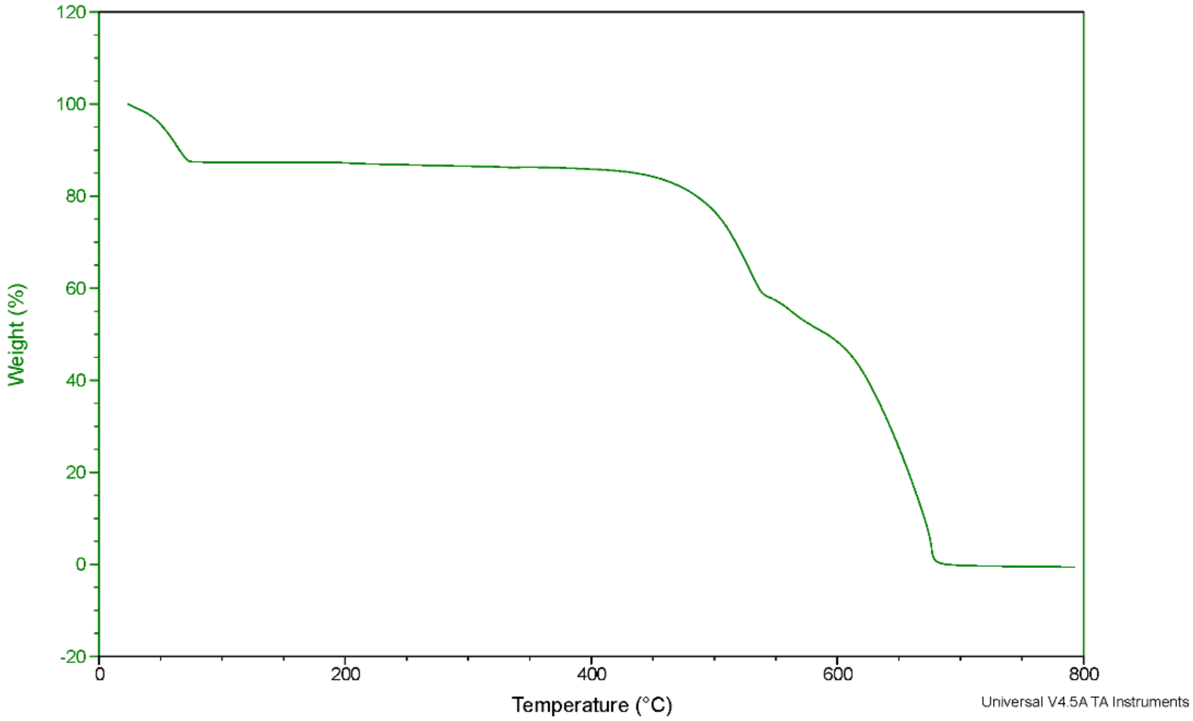

Figure S8. TGA curve under air of the as-synthesized sample of **M-TPY** crystallised in dichloromethane/ethanol, indicating a weight loss before 90 ºC due to the guest solvent removal.

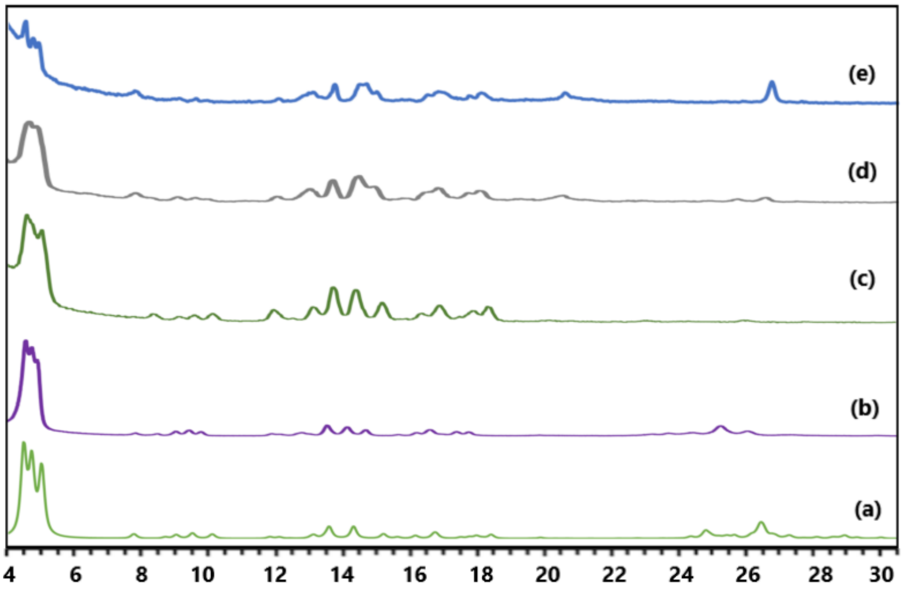

Figure S9. VT-PXRD patterns for **M-TPY**: (a) simulated from crystal data **M-TPY**, (b) heated at 140 °C, (c) 170 °C, (d) 200 °C and (e) 230 °C

a)

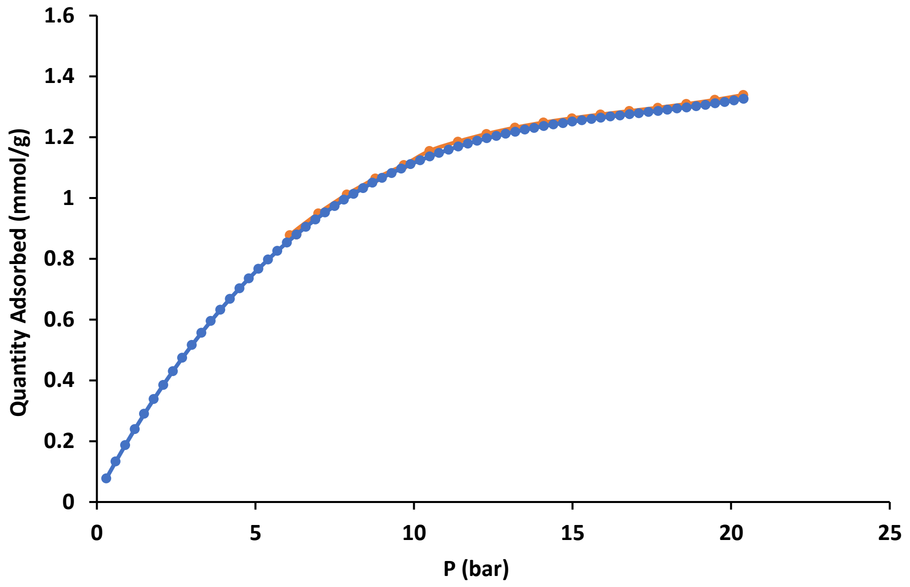

1.6
1.4
1.2
1
0.8
0.6
0.4
0.2
0
Quantity Adsorbed (mmol/g)
0
5
10
15
20
25
P (bar)

b)

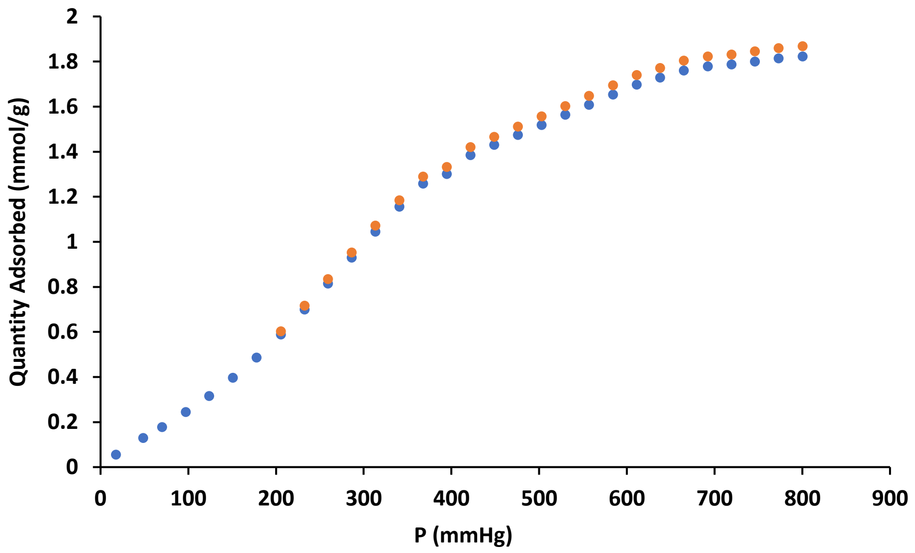

2
1.8
1.6
1.4
1.2
1
0.8
0.6
0.4
0.2
0
Quantity Adsorbed (mmol/g)
0
100
200
300
400
500
600
700
800
900
P (mmHg)

c)

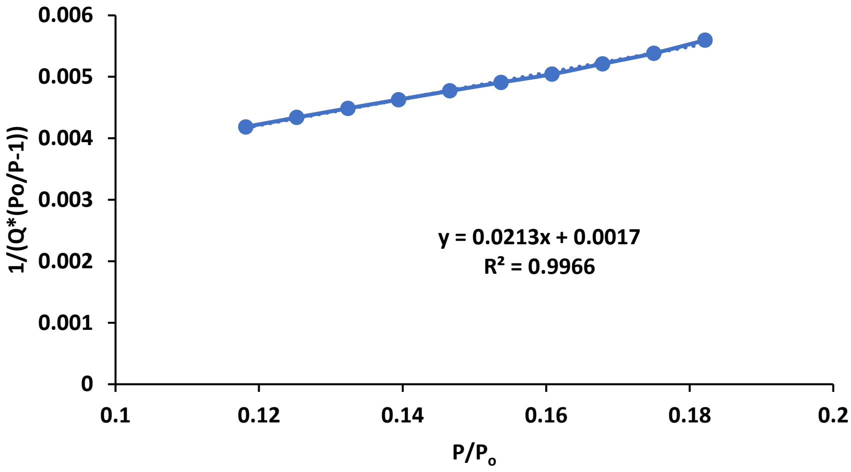

S_{BET}=((1/(0.0213 + 0.0017))/22414) × N_A ×A_m = 192m^2/g
A_m= cross-sectional area of CO_2
N_A = Avogrado's number

Figure S10. a) CO_2 adsorption isotherm for **M-TPY** at 273 K and b) 223K. Blue circles-adsorption; orange circles-desorption. c) Linear fit of data for **M-TPY** used to determine the BET surface area.

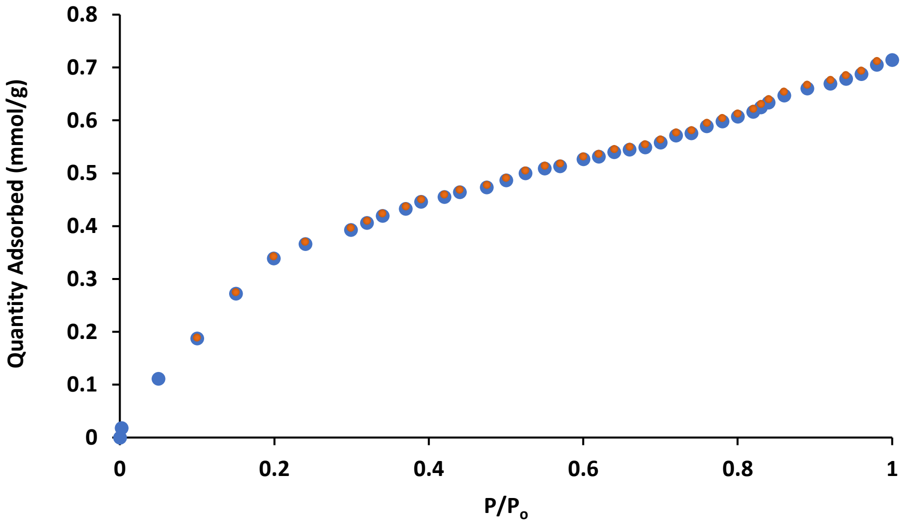

Figure S11. N_2 adsorption isotherm for **M-TPY** at 77 K. Blue circles-adsorption; orange circles-desorption

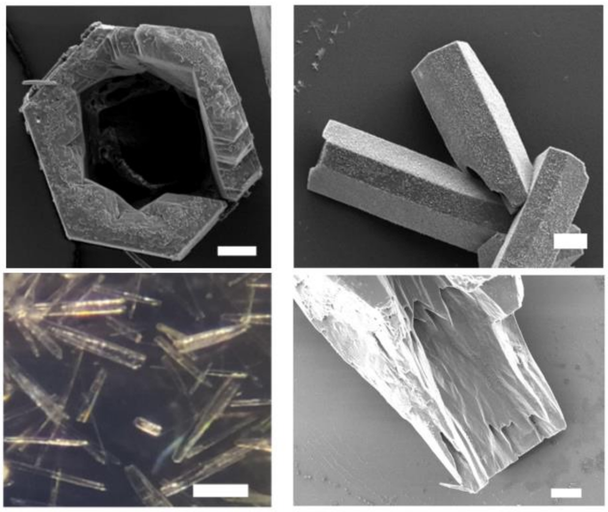

Figure S12. Additional SEM and optical microscopy images of crystals of **MM-TPY**. Scale bars: top left: 10 µm, top right: 10 µm, bottom left: 1 mm, bottom right: 10 µm.

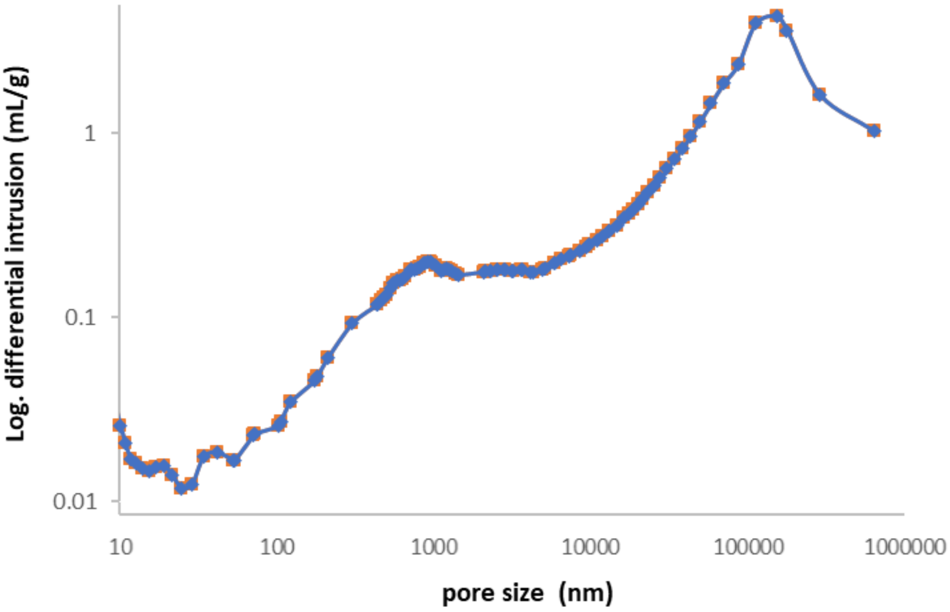

Figure S13. Pore size distribution for **MM-TPY** in logarithmic scale, determined by mercury intrusion porosimetry**.**

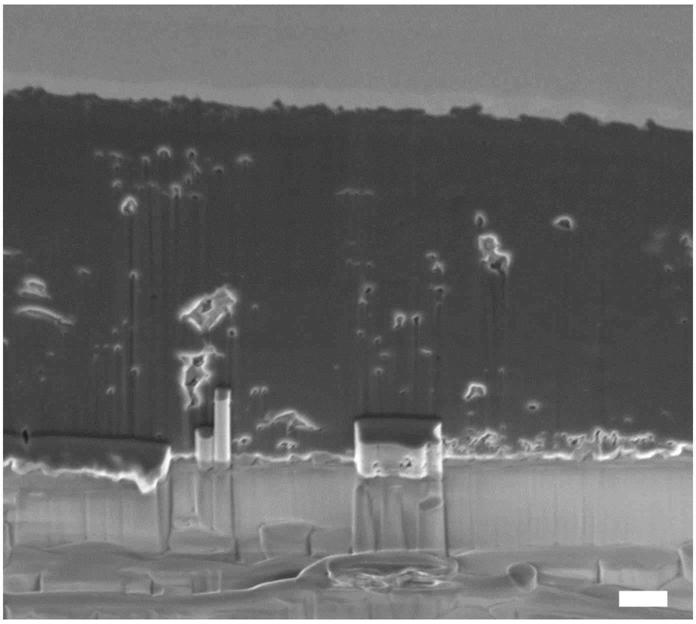

Figure S14. Cross-sectional area image of the solid part of the tubule of a **MM-TPY** crystal obtained by FIB-SEM. Scale bar: 1 μm.

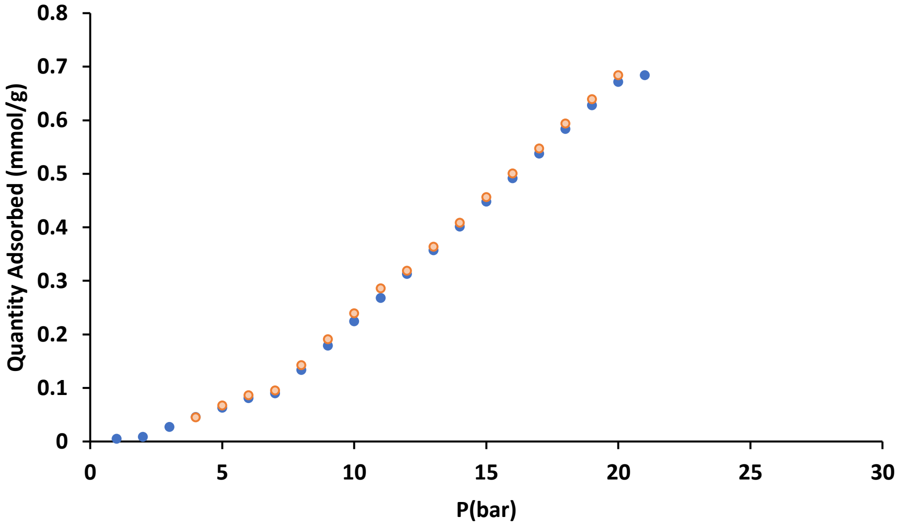

Figure S15. CO_2 adsorption isotherm for **MM-TPY** at 273 K. Blue circles-adsorption; orange circles-desorption.

a)

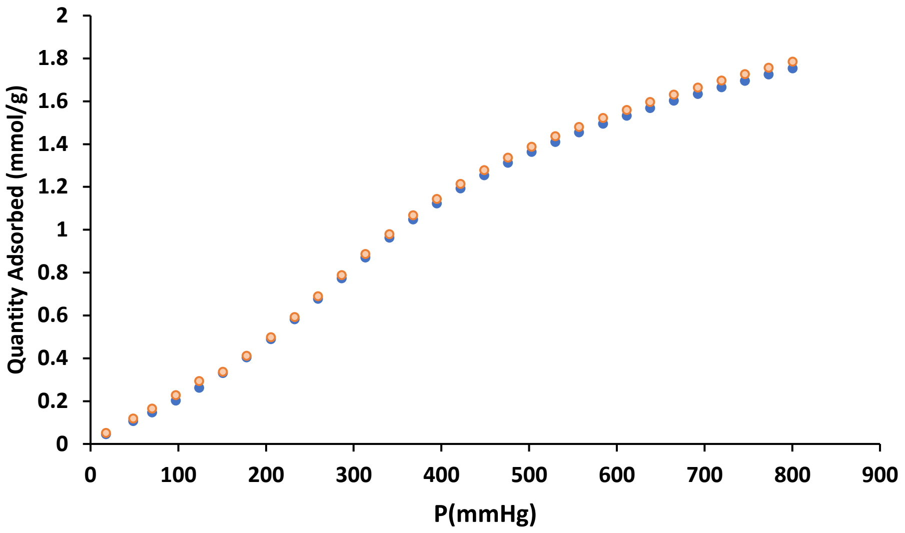

b)

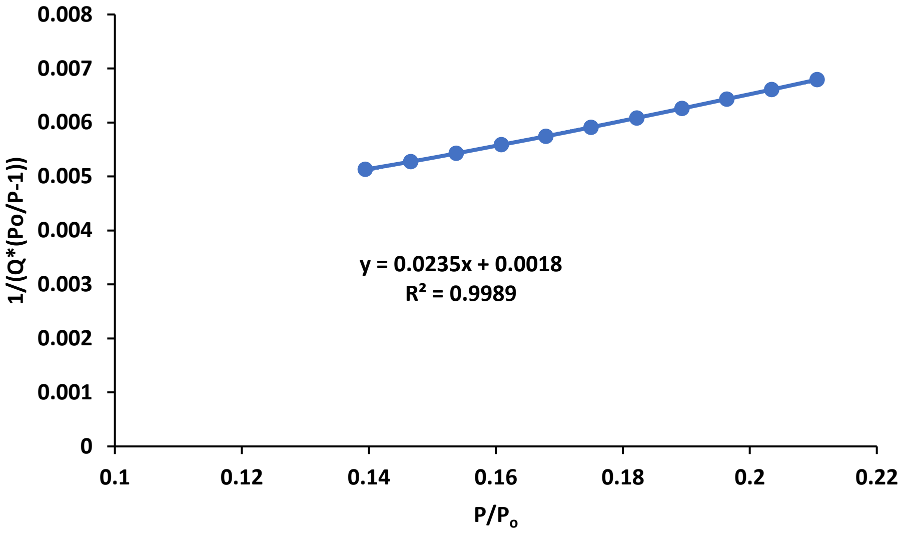

S_{BET}=((1/(0.0235 + 0.0018))/22414) × N_A ×A_m = 172 m^2/g
A_m= cross-sectional area of CO_2
N_A = Avogrado's number

Figure S16. a) CO_2 adsorption isotherm for **MM-TPY** at 223 K. Blue circles-adsorption; orange circles-desorption**. b)** Linear fit of data for **MM-TPY** used to determine the BET surface area.

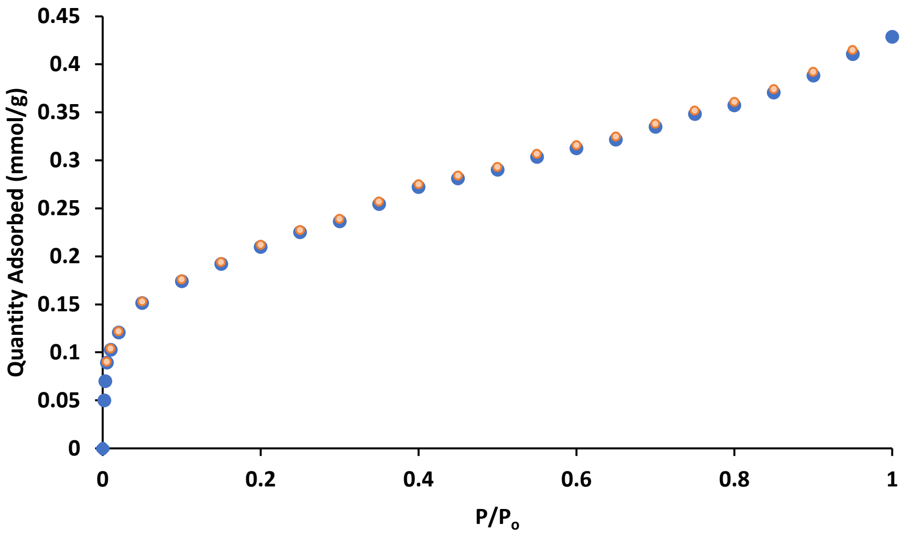

Figure S17. N_2 adsorption isotherm for **MM-TPY** at 77 K. Blue circles-adsorption; orange circles-desorption.

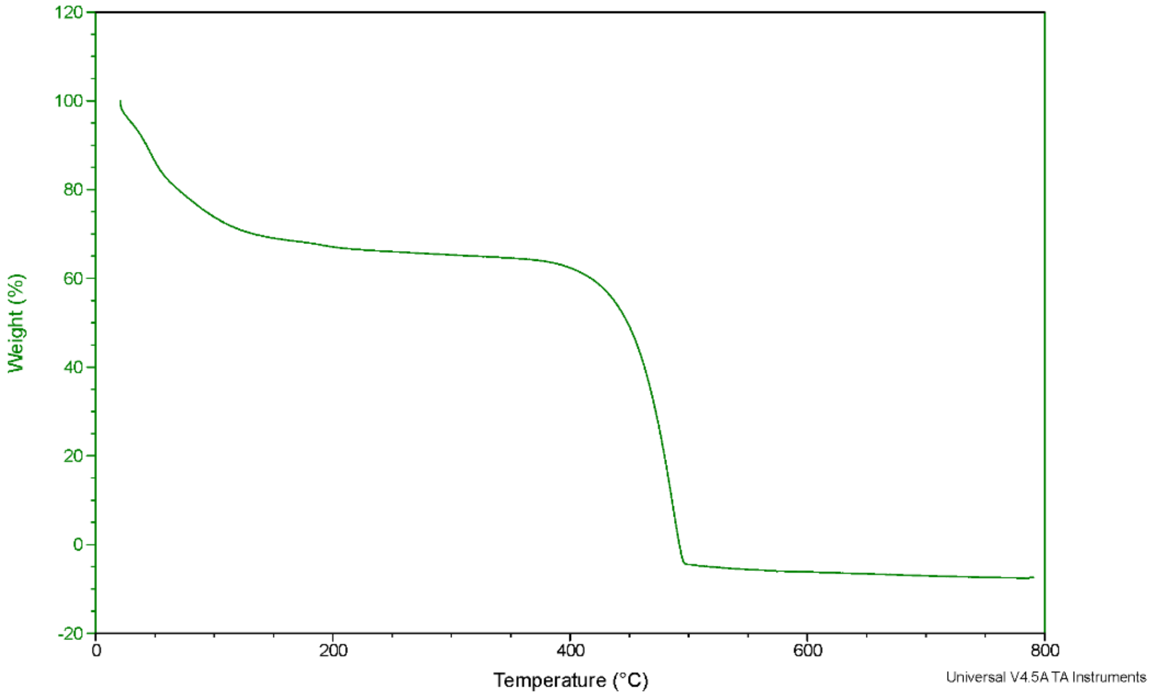

Figure S18. TGA curve under N_2 of the hierarchical tubular **MM-TPY** indicating a weight loss before 150 ^{o}C due to the guest solvent removal.

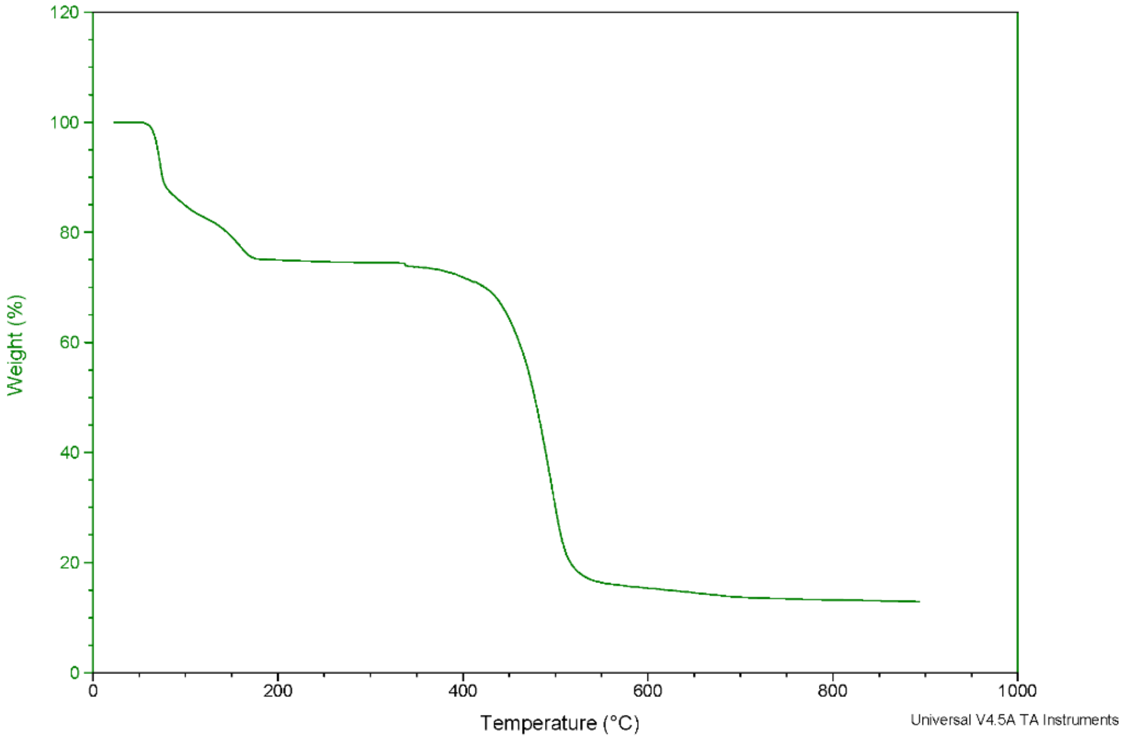

Figure S19. TGA curve under air of the hierarchical tubular **MM-TPY**, indicating a weight loss before 150 ^{o}C due to the guest solvent removal.

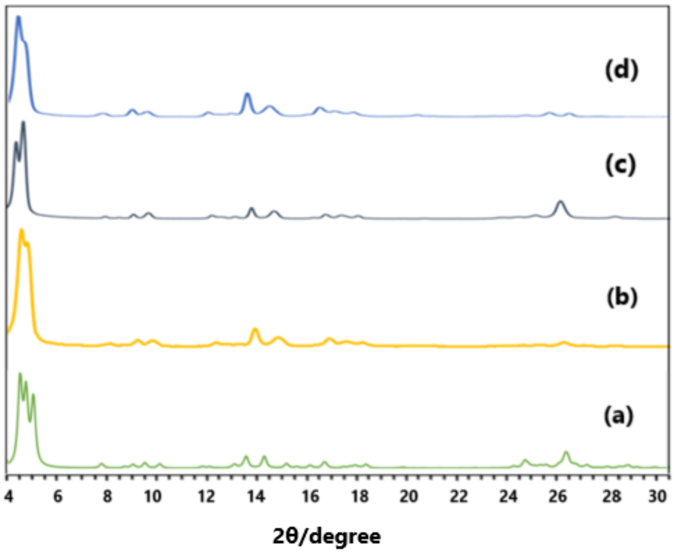

Figure S20. PXRD patterns for: (a) simulated from crystal data **M-TPY**, (b) as-synthesized **M-TPY**, (c) activated **M-TPY** and (d) activated **MM-TPY**

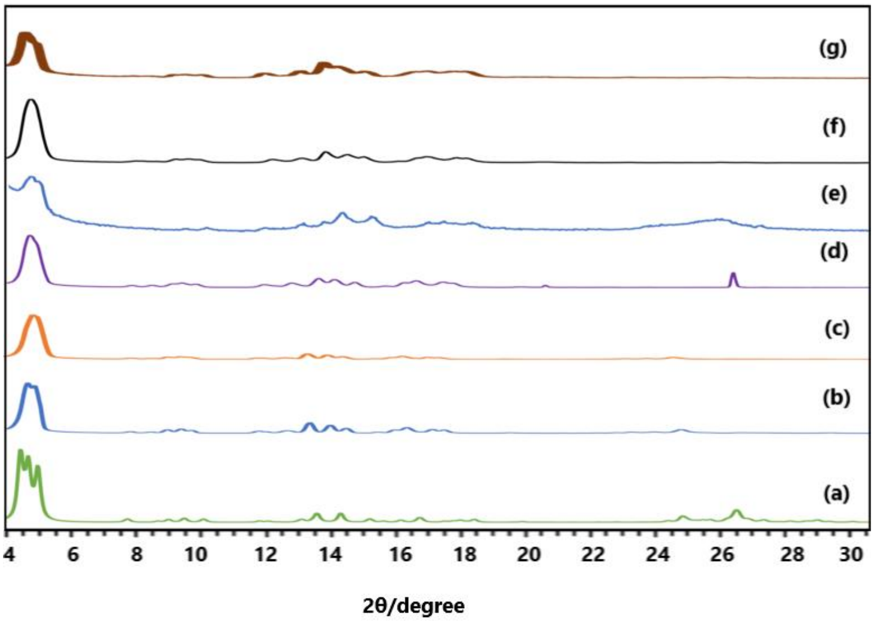

Figure S21. PXRD patterns for stability testing: (a) simulated from crystal data for **MM-TPY**, and after immersed in: (b) ethyl acetate, (c) water, (d) acetone, (e) DMF, (f) CH_3CN and (g) hexane.

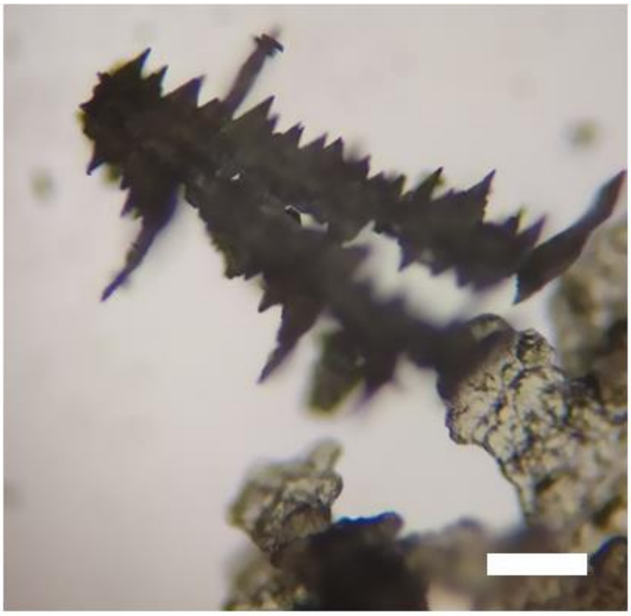

Figure S22. Optical microscopy image of crystallised **TPY** containing small impurities (<5% according ^{1}H NMR) derived from the synthesis. Scale bar: 1 mm.

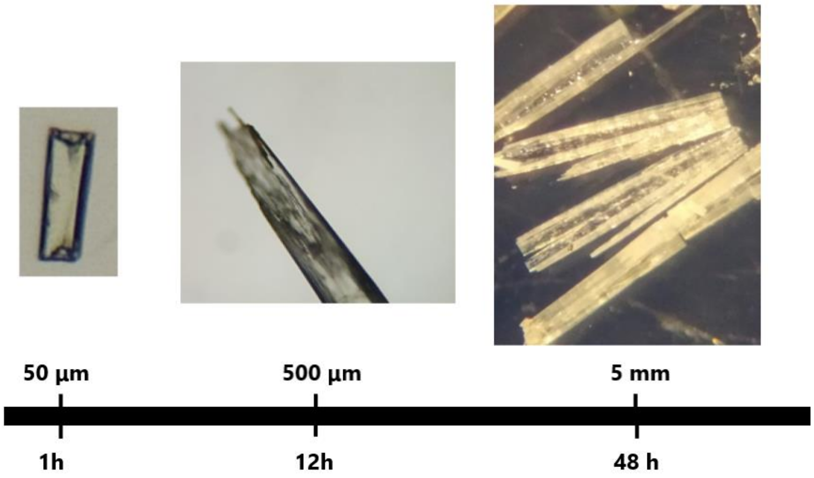

Figure S23. Evolution of **MM-TPY** crystal size over time by reducing the evaporation rate of the solvent.

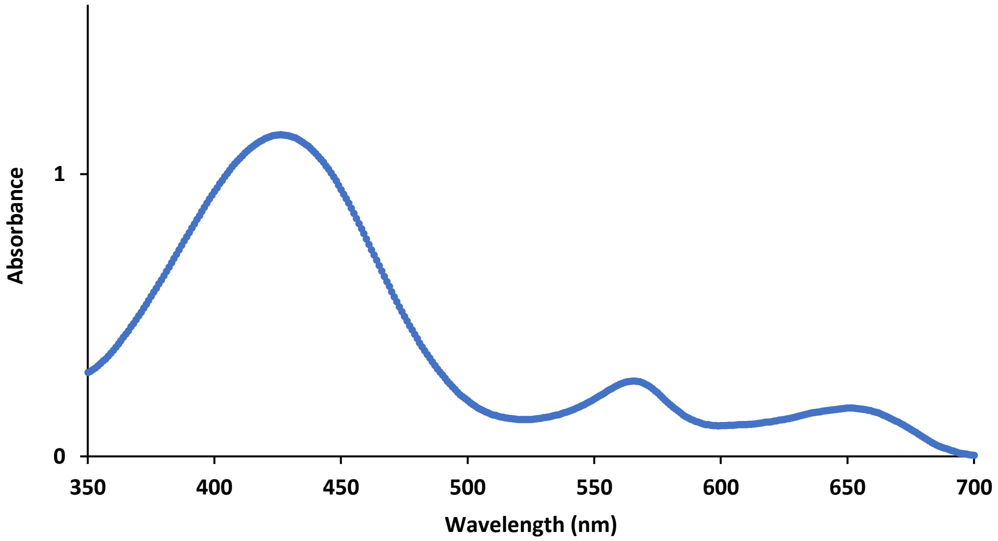

Figure S24. UV-Vis absorbance spectrum of dye mixture after desorption from **MM-TPY** crystals, using a solution of acetone/water 1:1 mixture.

a)

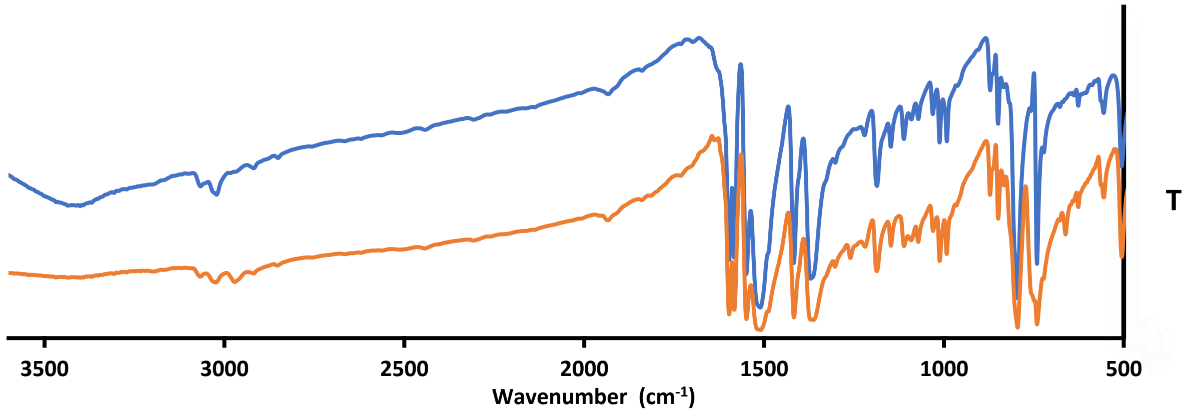

b)

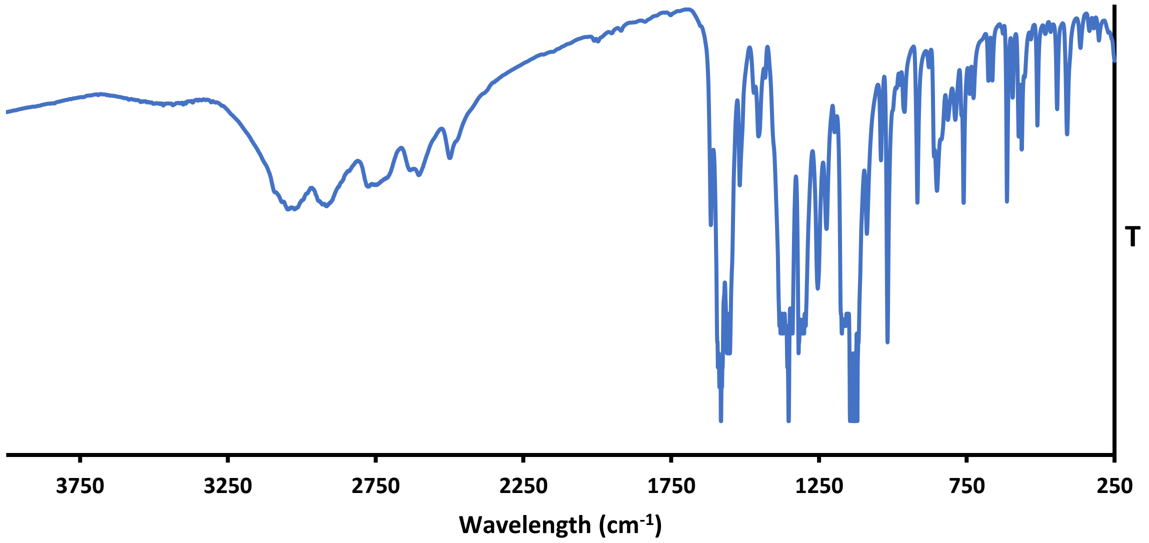

Figure S25. (a) FT-IR spectra of **MM-TPY** (blue), PR@**MM-TPY**(orange) and **(b)** FT-IR spectra of PR**.**

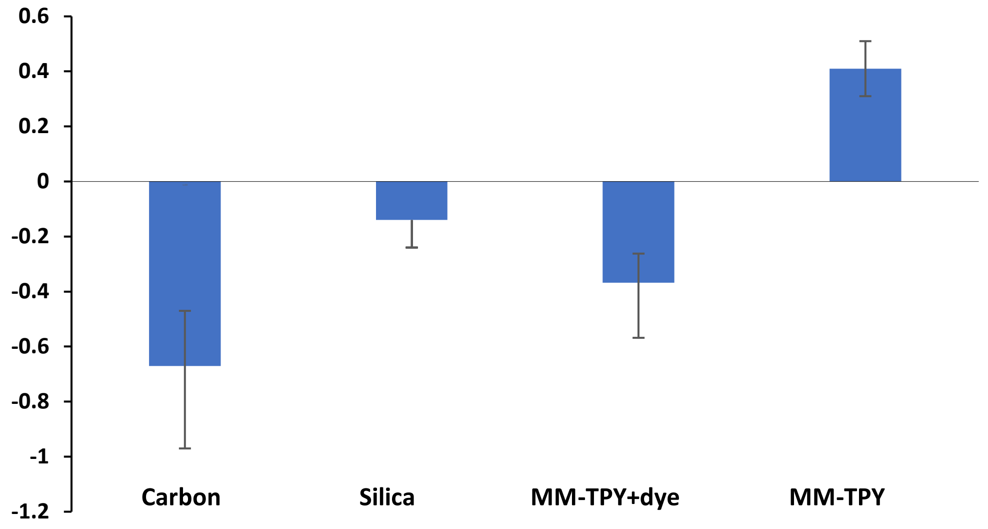

Figure S26. Zeta potential measurements from three replicated measurements for carbon and silica microparticles, **MM-TPY** loaded with PR dye (PR@**MM-TPY**) and **MM-TPY**.

References to Supplementary Information:

[1] J. Alvarez, G. Lopez, M. Amutio, J. Bilbao, M. Olazar, Upgrading the rice husk char obtained by flash pyrolysis for the production of amorphous silica and high quality activated carbon, *Bioresour. Technol.* **2014**, *170*, 132–137.

[2] Y. Zhao, D. G. Truhlar, The M06 suite of density functionals for main group thermochemistry, thermochemical kinetics, noncovalent interactions, excited states, and transition elements: two new functionals and systematic testing of four M06-class functionals and 12 other functionals, *Theor. Chem. Acc.* **2008**, *120*, 215–241

[3] P. C. Hariharan, J. A. Pople, The influence of polarization functions on molecular orbital hydrogenation energies, *Theor. Chim. Acta* **1973**, *28*, 213–222.

[4] N. Mardirossian, M. Head-Gordon, *ω*B97M-V: A combinatorially optimized, range-separated hybrid, meta-GGA density functional with VV10 nonlocal correlation, *J. Chem. Phys.* **2016**, *144*, 214110

[5] F. Weigend, R. Ahlrichs, Balanced basis sets of split valence, triple zeta valence and quadruple zeta valence quality for H to Rn: Design and assessment of accuracy, *Phys. Chem. Chem. Phys*. **2005**, *7*, 3297

[6] E. Epifanovsky, A. T. B. Gilbert, X. Feng, J. Lee, Y. Mao, N. Mardirossian, P. Pokhilko, A. F. White, M. P. Coons, A. L. Dempwolff, et al., Software for the frontiers of quantum chemistry: An overview of developments in the Q-Chem 5 package, *J. Chem. Phys.* **2021**, *155*, 084801.

Full list of references relevant to the manuscript:

[1] a) A. I. Cooper, Porous molecular solids and liquids, *ACS Cent. Sci.* **2017**, *3*, 544–553; b) M. A. Little, A. I. Cooper, The chemistry of porous organic molecular materials, *Adv. Funct. Mater*. **2020**, *30*, 1909842.

[2] S. Feng, Y. Shang, Z. Wang, Z. Kang, R. Wang, J. Jiang, L. Fan, W. Fan, Z. Liu, G. Kong, Y. Feng, S. Hu, H. Guo, D. Sun, Fabrication of a Hydrogen-Bonded Organic Framework Membrane through Solution Processing for Pressure-Regulated Gas Separation, *Angew. Chem. Int. Ed*. **2020**, *59*, 3840 –3845; *Angew. Chem.* **2020**,*132*, 3868 –3873.

[3] Q. Wang, D. Astruc, State of the Art and Prospects in Metal–Organic Framework (MOF)-Based and MOF-Derived Nanocatalysis, *Chem. Rev.* **2020**, *2*, 1438–1511.

[4] K. Geng, T. He, R. Liu, S. Dalapati, K. T. Tan, Z. Li. S. Tao, Y. Gong, Q. Jiang, D. Jiang, Covalent organic frameworks: design, synthesis, and functions, *Chem. Rev.* **2020**, *16*, 8814–8933.

[5] Z. Tang, X. Li, L. Tong, H. Yang, J. Wu, X. Zhang,T. Song, S. Huang, F. Zhu, G. Chen, G. Ouyang, A Biocatalytic Cascade in an Ultrastable Mesoporous Hydrogen-Bonded Organic Framework for Point-of-Care Biosensing, *Angew. Chem. Int. Ed*. **2021**, *60*, 23608 –23613; *Angew. Chem***.** **2021**,*133*, 23800 –23805.

[6] S. Yu, G.-L. Xing, L.-H. Chen, T. Ben, B.-L. Su, Crystalline porous organic salts: from micropore to hierarchical pores, *Adv. Mater*. **2020**, *32*, 2003270.

[7] T. Hasell, A.I. Cooper, Porous organic cages: soluble, modular and molecular pores, *Nat. Rev. Mater.* **2016**, *1*, 16053.

[8] Y. Tian, J. Yang, M. Gao, Z. Liu, M. Zhao, M. Fang, Z. Li, Organic microporous crystals driven by pure C–H··· π interactions with vapor-induced crystal-to-crystal transformations, *Mater. Horiz.* **2022**, *9*, 731–739.

[9] D. Menga, J. L. Yangc, C. Xiaod, R. Wanga, X. Xinge, O. Kocakf, G. Aydinf, I. Yavuzf, S. Nuryyevag, L. Zhangd, G. Liuh, Z. Lia, S. Yuani, Z.-Kui Wangi, W. Weij, Z. Wangh, K. N. Houkg, Y. Yanga, Noncovalent π-stacked robust topological organic framework, *Proc. Natl. Acad. Sci. U.S.A*. **2020**, *117*, 20397–20403.

[10] J. Tian, H. Wang, D.-W. Zhang, Y. Liu, Z.-T. Li, Supramolecular organic frameworks (SOFs): homogeneous regular 2D and 3D pores in water, *Natl. Sci. Rev.* **2017**, *4*, 426–436.

[11] G. Gong, S. Lv, J. Han, F. Xie, Q. Li,N. Xia, W. Zeng, Y. Chen, L. Wang, J. Wang, S. Chen, Halogen-Bonded Organic Framework (XOF) Based on Iodonium-Bridged N···I^+···N Interactions: A Type of Diphase Periodic Organic Network, *Angew. Chem. Int. Ed.* **2021**, *60*, 14831 –14835; *Angew. Chem.* **2021**,*133*, 14957 –14961.

[12] G. Zhang, B. Hua, A. Dey, M. Ghosh, B. A. Moosa, N. M. Khashab, Intrinsically porous molecular materials (ipms) for natural gas and benzene derivatives separations, *Acc. Chem. Res.* **2021**, *54*, 155−168.

[13 I. Hisaki, C. Xin, K. Takahashi, T. Nakamura, Designing hydrogen-bonded organic frameworks (HOFs) with permanent porosity, *Angew. Chem. Int. Ed.* **2019**, *58*, 11160–11170; *Angew.Chem.* **2019**,*131*,11278–11288.

[14] a) H. V. Doan, H. A. Hamzah, P. K. Prabhakaran, C. Petrillo, V. P. Ting Hierarchical Metal–Organic Frameworks with Macroporosity, *Nano-Micro Lett*. **2019**, 11:54; b) L. Feng, K.-Y. Wang, J. Willman, H.-C. Zhou, Hierarchy in Metal–Organic Frameworks, *ACS Cent. Sci.* **2020**, *6*, 359–367.

[15] W. Schwieger, A. G. Machoke, T. Weissenberger, A. Inayat, T. Selvam, M. Klumpp, A. Inayat, Hierarchy concepts: classification and preparation strategies for zeolite containing materials with hierarchical porosity, *Chem.Soc.Rev.* **2016**, *45*, 3353—3376.

[16] a) L. Feng, K.-Y. Wang, X.-L. Lv, T.-H. Yan, H.-C. Zhou, Hierarchically porous metal–organic frameworks: synthetic strategies and applications, *Natl. Sci. Rev.* **2020**, *7*, 1743–1758; b) L. Feng, K.-Y. Wang, J. Powell, H.-C. Zhou, Controllable synthesis of metal-organic frameworks and their hierarchical assemblies, *Matter* **2019,** *1*, 801-824.

[17] a) X. Zhao, P. Pachfule, S. Li, T. Langenham, M. Ye, X. Sclesiger, S. Paetz, J. Schmidt, A. Thomas, Macro/Microporous Covalent Organic Frameworks for Efficient Electrocatalysis, *J. Am. Chem. Soc.* **2019**, *141,* 6623−6630; b) R.-R. Liang, S.-Yan, J. R.-Han, A. X. Zhao, Two-dimensional covalent organic frameworks with hierarchical porosity, *Chem. Soc. Rev*. **2020**, *49*, 3920.

[18] K. Shen, L. Zhang, X. Chen, L. Liu, D. Zhang, Y. Han, J. Chen, J. Long, R. Luque, Y. Li, B. Chen, Ordered macro-microporous metal-organic framework single crystals, *Science* **2018**, *359*, 206–210.

[19] a) Q. Yin, Y.-L. Li, L. Li, J. Lü, T.-F. Liu, R. Cao, Novel Hierarchical Meso-Microporous Hydrogen-Bonded Organic Framework for Selective Separation of Acetylene and Ethylene versus Methane, *ACS Appl. Mater. Interfaces* **2019**, *11*, 17823−17827; b) L. K. Shrestha, Y. Yamauchi, J. P. Hill, K. Miyazawa, K. Ariga, Fullerene Crystals with Bimodal Pore Architectures Consisting of Macropores and Mesopores, *J. Am. Chem. Soc.* **2013**, *135*, 586–589; c) M. Hua, S. Wang, Y. Gong, J. Wei, Z. Yang, J.-Ke Sun, Hierarchically Porous Organic Cages, *Angew. Chem. Int. Ed.* **2021**, *133*, 12490–12497; *Angew. Chem.* **2021**, *133*, 12598–12605.

[20] H. Yamagishi, H. Sato, A. Hori,Y. Sator, O. Matsuda, K. Kato, T. Aida, Self-assembly of lattices with high structural complexity from a geometrically simple molecule, *Science* **2018**, *361*, 1242–1246.

[21] Cambridge Crystallographic Data Centre, CCDC 2164359

[22] a) P.V.D. Sluis, A.L. Spek, BYPASS: an effective method for the refinement of crystal structures containing disordered solvent regions, *Acta Crystallogr*. **1990**, *A46*, 194-201; b) A.L. Spek, PLATON SQUEEZE: a tool for the calculation of the disordered solvent contribution to the calculated structure factors, *Acta Crystallogr. Sect. C-Struct. Chem*. **2015**, *71*, 9-18.

[23] P.R. Spackman, M. J. Turner, J. J. McKinnon, S. K. Wolff, D. J. Grimwood, D. Jayatilaka, M. A. Spackman, *CrystalExplorer*: a program for Hirshfeld surface analysis, visualization and quantitative analysis of molecular crystals, *J. Appl. Cryst.* **2021**, *54*, 1006–1011.

[24] P. Wei, X. He, Z. Zheng, D. He,Q. Li, J. Gong, J. Zhang, H. H. Y. Sung, I. D. Williams, J. W. Y. Lam, M. Liu, B. Z. Tang, Robust Supramolecular Nano-Tunnels Built from Molecular Bricks, *Angew. Chem. Int. Ed.* **2021**, *60*, 7148 – 7154; *Angew. Chem.* **2021**, *133*, 7224–7230.

[25] a) B.-T. Liu, X.-H. Pan, D.-Y. Zhang, R. Wang, J.-Y. Chen, H.-R. Fang, T.-F. Liu, Construction of Function-Oriented Core–Shell Nanostructures in Hydrogen-Bonded Organic Frameworks for Near-Infrared-Responsive Bacterial Inhibition, *Angew. Chem. Int. Ed.* **2021**, *60*, 25701–25707; *Angew. Chem.* **2021**,*133*, 25905 –25911; b) Q. Huang, W. Li, Z. Mao, L. Qu, Y. Li, H. Zhang, T. Yu, Z. Yang, J. Zhao, Y. Zhang, M. P. Aldred, Z. Chi, An exceptionally flexible hydrogen-bonded organic framework with large-scale void regulation and adaptive guest accommodation abilities, *Nat. Comm.* **2019**, *10*, 3074.

[26] Z. Yang, J. Zhang, L. Zhang, B. Fu, P. Tao, C. Song, W. Shang, T. Deng, Self-Assembly in Hopper-Shaped Crystals, *Adv. Funct. Mater.* **2020**, *30*, 1908108.

[27] M. Li, C. Zhang, M. Li, F. Liu, L. Zhou, Z. Gao, J. Sun, D. Han, J. Gong, *Growth defects of organic crystals: A review*, *Chem. Eng. J.* **2022**, *429*, 132450.

[28] a) A. G. Zavyalova, D. V. Kladko, I. Y. Chernyshov, V. V. Vinogradov, Large MOFs: synthesis strategies and applications where size matters, *J. Mater. Chem. A* **2021**, *9*, 25258-25271; b) P. Falcaro, K. Okada, T. Hara, K. Ikigaki, Y. Tokudome, A. W. Thornton, A. J. Hill, T. Williams, C. Doonan, M. Takahashi, Centimetre-scale micropore alignment in oriented polycrystalline metal–organic framework films via heteroepitaxial growth, *Nat. Mater.* **2017,** *16*, 342–348.

[29] a) Q. Tang, S. Maji, B. Jiang, J. Sun, W. Zhao, J. P. Hill, K. Ariga, H. Fuchs, Q. Ji, L. K. Shrestha, Manipulating the Structural Transformation of Fullerene Microtubes to Fullerene Microhorns Having Microscopic Recognition Properties, *ACS Nano* **2019**, *13*, 14005−14012; b) P. Bairi, K. Minami, J. P. Hill, K. Ariga, L. K. Shrestha, Intentional Closing/Opening of “Hole-in-Cube” Fullerene Crystals with Microscopic Recognition Properties, *ACS Nano* **2017**, *11*, 7790–7796.

[30] X.-Y. Liu, F. Zhang, T.-W. Goh, Y. Li, Y-C. Shao, L. Luo, W. Huang, Y.-T. Long, L.-Y. Chou, C.-K. Tsung, Using a Multi-Shelled Hollow Metal-Organic Framework as a Host to Switch the Guest-to-Host and Guest-to-Guest Interactions,*Angew. Chem. Int. Ed.* **2018**, *57*, 2110–2114; Angew. Chem. **2018**, 130, 2132 –2136.

[31] a) X. Xie, X. Huang, W. Lin, Y. Chen, X. Lang, Y. Wang, L. Gao, H. Zhu, J. Chen, Selective Adsorption of Cationic Dyes for Stable Metal–Organic Framework ZJU-48, *ACS Omega* **2020**, *5*, 13595−13600; b) C. Huang, Z. Guo, X. Zhen, X. Chen, Z. Xue, S. Zhang, X. Li, B. Guan, X. Li, G. Hu, T. Wang, Deformable Metal−Organic Framework Nanosheets for Heterogeneous Catalytic Reactions, *J. Am. Chem. Soc.* **2020**, *142*, 9408–9414.

[32] S. G.-Guerrero, N. Otero, M. Queizán, M. M. Alonso, Potential Application of h-BNC Structures in SERS and SEHRS Spectroscopies: A Theoretical Perspective, *Sensors* **2019**, *19*, 1896.

[33] N. Cao, Y. Zhang, L. Chen, W. Chu, Y. Huang, Y. Jia, M. Wang, An innovative approach to recover anode from spent lithium-ion battery, *J. Power Sources* **2021**, *31*, 229163.

[34] T. Salafi, K. K. Zeming, Y. Zhang, Advancements in microfluidics for nanoparticle separation, *Lab on Chip* **2017**, *17*, 11-33.

[35] F. Schoden, M. Dotter, D. Knefelkamp, T. Blachowicz, E. S. Hellkamp, Review of State of the Art Recycling Methods in the Context of Dye Sensitized Solar Cells, *Energies* **2021**, *14*, 3741.
